# The Impact of Daytime Napping Following Normal Night-Time Sleep on Physical Performance: A Systematic Review, Meta-analysis and Meta-regression

**DOI:** 10.1007/s40279-023-01920-2

**Published:** 2023-09-12

**Authors:** Omar Boukhris, Khaled Trabelsi, Haresh Suppiah, Achraf Ammar, Cain C. T. Clark, Haitham Jahrami, Hamdi Chtourou, Matthew Driller

**Affiliations:** 1 SIESTA Research Group, School of Allied Health, Human Services and Sport, La Trobe University, Melbourne, Australia; 2 High Institute of Sport and Physical Education, University of Sfax, Sfax, Tunisia; 3 Research Laboratory, Education, Motricity, Sport and Health (EM2S), LR15JS01, High Institute of Sport and Physical Education, University of Sfax, Sfax, Tunisia; 4 Department of Training and Movement Science, Institute of Sport Science, Johannes Gutenberg-University Mainz, Mainz, Germany; 5 Centre for Intelligent Healthcare, Coventry University, Coventry, UK; 6 Department of Psychiatry, Ministry of Health, Manama, Bahrain; 7 Department of Psychiatry, College of Medicine and Medical Sciences, Arabian Gulf University, Manama, Bahrain; 8 Physical Activity, Sport, and Health, UR18JS01, National Observatory of Sport, Tunis, Tunisia; 9 Sport, Performance, and Nutrition Research Group, School of Allied Health, Human Services and Sport, La Trobe University, Melbourne, Australia

## Abstract

**Background:**

Daytime napping is used by athletes as a strategy to supplement night time sleep and aid physical performance. However, no meta-analytical overview regarding the impact of napping following a night of normal sleep (7–9 h) on physical performance is available.

**Objective:**

The aim of this study was to evaluate the effect of daytime napping following normal night-time sleep on physical performance in physically active individuals and athletes.

**Methods:**

This systematic review and meta-analysis was conducted in accordance with the Preferred Reporting Items for Systematic Reviews and Meta-Analysis (PRISMA) guidelines. Seven electronic databases (i.e., PubMed, Web of Science, Scopus, SPORTDiscus, CINAHL, SCIELO, and EBSCOhost) were used to search for relevant studies that investigated the impact of daytime napping, following normal night-time sleep, on physical performance in physically active individuals and athletes, published in any language, and available before September 01, 2022. Studies that included assessments of any physical performance measures were included. QualSyst was used to assess the methodological quality of the studies.

**Results:**

Of 18 selected articles, 15 were of strong quality and 3 were of moderate quality. Compared with no-nap conditions, physically active individuals and athletes who napped experienced an increase in highest distance (effect size [ES] 1.026; *p* < 0.001) and total distance (ES 0.737; *p* < 0.001), and a decrease in fatigue index (ES 0.839, *p* = 0.008) during the 5-m shuttle run test (5MSRT). However, napping yielded no effect on muscle force (ES 0.175; *p* = 0.267). No effect of napping was found in one study that measured sprint performance and in two studies that measured performance during the 30-s Wingate test. Two of three studies reported an increase in jump performance after napping. Two of three studies reported an increase in repeated sprints after napping. One study reported an increase in upper-body power performance after napping, and napping was beneficial for endurance performance in one of two studies.

**Conclusion:**

Following normal sleep, napping is beneficial for the performance of the 5MSRT, with no significant effect on muscle force. No firm conclusions can be drawn regarding other physical performance measures due to the limited number of studies.

**Supplementary Information:**

The online version contains supplementary material available at 10.1007/s40279-023-01920-2.

## Key Points

**Table:** 

Daytime napping before afternoon training sessions and/or competition could be recommended as a way to supplement night-time sleep as well as enhance athletic performance.
Longer naps might be more beneficial in optimizing physical performance.
The impact of a diurnal nap may be affected by (i) nap durations, (ii) time of day of naps, (iii) sleep inertia, and (iv) exercise type.

## Introduction

In order to attain peak performance, optimization of the recovery process is important, with sleep being one of the crucial components, especially for athletes [1]. In this context, it has been suggested that athletes may need a greater sleep duration than the general population because of augmented physical and mental demands on their bodies, resulting from repeated exposure to competition and high-intensity training [2]. Indeed, while healthy adults are encouraged to sleep 7–9 h per night [3], it has been recommended that athletes obtain 9–10 h of sleep per night for optimal recovery [4]. Nevertheless, due to several factors, such as training early in the morning, late-night competition, bright light exposure at night, jetlag, high training loads, and/or disturbed sleep before a competition, athletes often do not have adequate sleep quality and quantity [5–7]. Therefore, while night-time sleep is often curtailed in athletes, they may seek to use napping as a strategy to further complement their night-time sleep.

Importantly, human performance (i.e., tasks that require concentration, alertness, and attention, as well as physical tasks that require speed and muscle strength) tends to be reduced in response to the post-lunch dip phenomenon [8–10], which occurs between 13:00 h and 16:00 h, due to an increase in the tendency to sleep and decreases in core temperature and vigilance [11]. Therefore, daytime napping is regarded as a recovery strategy often used to counteract impaired performances as a consequence of the post-lunch dip [12]. Moreover, as athletes have been recommended to get 9–10 h of sleep per night [4], daytime napping could be used as a prophylactic supplement to a full night’s sleep to achieve peak performances. The nap, as a performance/recovery tool, has piqued the interest of sports science researchers as it has several positive effects, especially in recovery and boosting physical performance [11–14]. In this context, Chtourou et al. [11] concluded that daytime napping following normal sleep was beneficial in improving physical performance during the 5-m shuttle run test (5MSRT). Furthermore, according to a narrative review by Botonis et al. [12], a diurnal nap could improve physical performance after a full night's sleep and could also be a strategy for maintaining physical performance when sleep loss is faced. Recently, two systematic reviews on the effect of daytime napping on physical performance [13, 14] recommended napping to enhance physical performance following sleep deprivation or even after a night of normal sleep. Furthermore, the authors suggested how certain factors, such as the previous night’s sleep, sleep inertia, nap duration, and/or exercise type, could influence the effect of napping on physical performance [13, 14].

Nevertheless, firm conclusions cannot be drawn from these narrative and systematic reviews as, to our knowledge, no meta-analytical overview regarding the impact of napping on physical performance is available. Therefore, a meta-analysis is needed to quantitatively synthesize the results of pooled studies, potentially permitting more meaningful insights with a higher level of evidence compared with systematic reviews [15, 16]. In addition, optimization of sleep is considered an imperative element for athletes, with sleep extension potentially improving athletic performance [2, 17]. Therefore, it is worthwhile to gain a better understanding from studies that examined the impact of napping following a full night’s sleep, rather than those that have investigated the use of napping after sleep deprivation or restriction.

Therefore, the purpose of this paper was to systematically review the expanding evidence base and, where possible, conduct meta-analyses to investigate the effects of daytime napping following normal night-time sleep (e.g., not sleep restricted or deprived) on athletic performance. We hypothesized that daytime napping following normal night-time sleep would enhance physical performance in physically active individuals and athletes.

## Methods

### Protocol

This systematic review was conducted in accordance with the Preferred Reporting Items for Systematic Reviews and Meta-Analysis (PRISMA) guidelines [18, 19]. A protocol was created in advance and is available upon request from the corresponding author.

### Eligibility Criteria

Peer-reviewed journal articles, written in any language, that examined the impact of daytime napping on athletes (i.e., individuals who train regularly ~ 3 times per week with the purpose to completed [20]) or physically active individuals (i.e., those who complete﻿d at least 150–300 min moderate-intensity activity or 75–150 min of vigorous-intensity activity a week for health, fitness, or recreational purposes [20]), were considered. Descriptive or review articles, conference proceedings, and articles based on sleep deprivation or sedentary individuals or without physical exercise performed after napping were excluded. However, no restrictions were applied in terms of study design, setting, country, or time frame. Assessments examining physical performance were included.

### Information Sources and Search

Seven electronic databases (PubMed, Web of Science, Scopus, SPORTDiscus, CINAHL, SCIELO, and EBSCOhost) were searched, without applying any time limits or filters, using the following keywords: [(nap) OR (napping) OR (daytime nap) OR (daytime sleep) OR (siesta)] AND [(physically active) OR (physical activity) OR (athletes)] AND [(sports) OR (sport) OR (performance) OR (athletic performance) OR (physical functional performance) OR (physical performance) OR (jump performance) OR (repeated sprint) OR (sprint) OR (sprint performance) OR (speed) OR (muscle strength) OR (strength) OR (anaerobic performance) OR (aerobic performance) OR (power) OR (physical endurance) OR (endurance) OR (exercise) OR (high-intensity exercise) OR (repeated high-intensity exercise)]. Wild-card options (i.e., truncated words) and medical subject heading (MeSH) terms were also used where appropriate. Searches were completed on September 01, 2022. In addition, a review of the reference lists of included studies, as well as citations from other journals, identified via Google Scholar, was undertaken. Specialists in the field were also contacted for information on possible upcoming studies. Furthermore, specific target journals (i.e., Journal of Sports Sciences, Biological Rhythm Research, International Journal of Sport Physiology and Performance, British Journal of Sports Medicine, European Journal of Sport Sciences, Sleep Medicine, Sleep, International Journal of Environmental Research and Public Health, Sports, Chronobiology International, Journal of Sleep Research, Asian Journal of Sports Medicine) were hand-searched for relevant manuscripts. Details on the search strategy used are provided in Table S1 of the electronic supplementary material (ESM).

### Study Selection

**Fig. 1:**
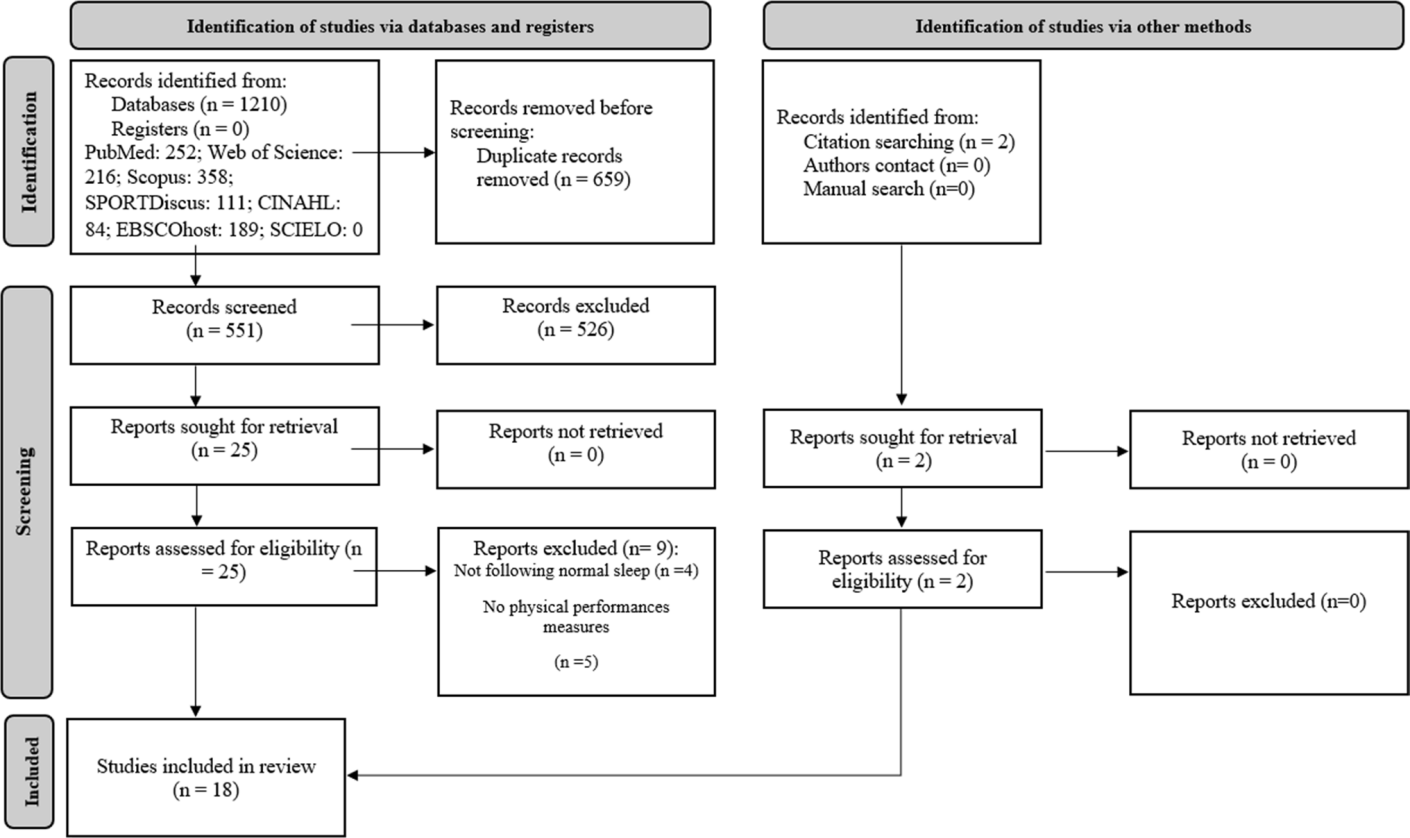
PRISMA flow diagram

The process for selecting articles is summarized in Fig. 1. Zotero was used in order to eliminate duplicate articles recorded in the initial search. Screening of titles and abstracts of all unique hits were conducted by two authors (OB and KT) for eligibility, and disagreements were resolved by consensus. Selected articles were then reviewed fully for the purpose of finalizing eligibility or exclusion, and reasons for exclusion were cited.

### Data Collection Process

A pilot-tested extraction form was used in order to collect data by two authors (OB and KT), and disagreements were resolved by consensus. Participant characteristics (number, sex, age, level of practice, and activity), study characteristics (nap duration, time of day of napping, time between the end of napping and the exercise), and key findings were included in the data extracted.

### Quality Assessment

QualSyst was utilized as an assessment tool for the methodological quality of each study [21]. A 3-point scale (yes = 2, partial = 1, no = 0) was used to rate the 14 items included in QualSyst. ‘NA’ was marked for items that were not applicable to a particular study design. Each article had a summary score, which was based on the total relevant items divided by the total possible score. The assessment of studies was conducted by two authors (OB and KT), and disagreements over ratings were solved by discussion or by the intervention of a third author (MD) when necessary. Studies were considered of strong quality if they scored ≥ 75%, moderate quality if they scored 55%–75%, and weak quality if they scored ≤ 55%. The proportion of lost points for each item was also calculated.

### Meta-analysis

The commercial software Comprehensive Meta-Analysis (CMA for Windows, version 3, Biostat, Englewood, NJ 2013, USA) was utilized for the purpose of conducting a meta-analysis. Random-effects meta-analysis models were used. According to Cohen, effect sizes (ES) with 95% confidence intervals (CI) were determined, reflecting standardized differences in means between no-nap condition and nap condition for physical performance, that is, highest distance (HD) (i.e., the greatest distance covered during a 30-s shuttle), total distance (TD) (i.e., the total distance covered during the six 30-s shuttles), fatigue index (FI) during 5MSRT, and muscle force. Effect sizes were considered extremely large (ES > 4.0), very large (ES > 2.0), large (ES 1.2–2.0), moderate (ES 0.6–1.2), small (ES 0.2–0.6), and trivial (ES < 0.2) [22]. Statistical heterogeneity was assessed by Q [23] and *I*^2^ [24]. Evidence of substantial heterogeneity was considered when the *I*^2^ value was > 50% [24]. *I*^2^ value was rated as low (25%), moderate (50%), and high (75%) statistical heterogeneity [24].

When an article contained a control group (i.e., no-nap) and more than one nap group (i.e., nap duration), we separately labeled each nap group and divided the sample size of the control group by the number of nap groups [25].

Further stratification, related to the most important characteristics, was conducted to identify potential sources of variance and heterogeneity; meta-regression analyses investigated quantitative relationships between dependent variables and covariates. Moderators included population size, age, level of practice, activity, nap duration, time of day of napping, and time between the end of napping and the exercise.

The stability of the pooled ES was assessed by sensitivity analyses computing the impact of excluding individual studies from the analysis. In addition, in order to confirm the stability and reliability of the results, a cumulative meta-analysis, which aims to aggregate accumulating evidence with additional studies based on their chronological order, was executed to further ensure the stability and reliability of the results. Funnel plots examined probable publication bias, seeking possible asymmetries on visual inspection, and performing Begg and Mazumdar’s rank correlation test (Kendall’s *S* statistic P–Q) [26], Egger’s linear regression test [27], and Duval and Tweedie’s trim-and-fill test [28]. A significance level of *p* < 0.05 was adopted for all analyses.

## Results

### Study Selection

The initial search resulted in 1210 individual records, of which 551 remained after excluding duplicates. Then, 25 published articles remained after screening titles and abstracts (Fig. 1). After a careful review of the 25 full texts, 16 articles were included. A review of reference lists and related citations identified via Google Scholar added two further appropriate articles, yielding a total of 18.

### Study Characteristics

A total of 18 studies, comprising 269 participants, were included in this meta-analysis. The studies were published between the years 2014 and 2022. The characteristics of the 18 studies are presented in Table 1. The highest number of participants was 27, in the study of Pelka et al. [29]; numbers ranged between seven and 27 in the remaining reports. The average age of participants ranged from 15 to 35 years. Of all the studies reviewed, only O'Donnell et al. [30] included female athletes; the remaining studies focused exclusively on male participants. The study population included physically active participants who were considered moderately trained in five studies, and trained athletes in 13 studies. Included studies focused on the acute impacts of daytime napping on physical performance (i.e., 5MSRT, muscle force, sprint performance, jump performance, 30-s Wingate test, repeated sprint, and endurance performance).

**Table 1:** A summary of the 18 studies assessing physical performance after napping following normal sleep in physically active and athletes individuals

Study	Country	Sample size and sex	Age (years)	Level of practice	Activity	Sleep duration for the night before each condition (method of sleep measurement)	Nap duration (min)	Time of day of napping	Time between end of napping and exercise (min)	Method of sleep measurement	Measured parameters	Results
Petit et al. [43]	France	16 male	22 ± 2	Athletes	NM	≈ 8 h (polysomnography)	20	1300 h	210	Polysomnography	Peak power (watts) during Wingate test	NS
											Mean power (watts) during Wingate test	NS
											FI (%) during Wingate test	NS
Pelka et al. [29]	Germany	27 male	25 ± 1	Athletes	Individual sports (e.g., track and field, tennis) (*n* = 12) and team sports (e.g., football, handball) (*n* = 15)	No sleep restriction (self-report)	25	NM	NM	None	Average maximum speed of the 6 × 4 s sprints (m/s)	↑ by 0.76% after nap vs no nap
Blanchfield et al. [46]	United Kingdom	11 male	35 ± 12	Athletes	Individual sport (i.e., running)	≈ 7 h (actigraphy)	40	NM	NM	Actigraphy	Running time to exhaustion at 90% $\dot{V}$O_2max_ (s)	↑ running time to exhaustion after nap for 5 runners with < 7–9 h night-time sleep
O'Donnell et al. [30]	New Zealand	14 female	23 ± 6	Athletes	Team sport (i.e., netball)	No sleep restriction (self-report)	< 20	1330 h	30	None	Peak jump velocity (m·s^−1^)	↑ by 4.95% after short nap vs no nap
											Mean jump velocity (m·s^−1^)	NS
											Jump height (cm)	NS
							> 20				Peak jump velocity (m·s^−1^)	NS
											Mean jump velocity (m·s^−1^)	↑ by 3.42% after long nap vs no nap
											Jump height (cm)	NS
Tanabe et al. [38]	Japan	7 male	21 ± 4	Physically active	NM	≈ 8 h (polysomnography)	30	1330 h	60	Polysomnography	Grip strength (N)	NS
											Back strength (N)	NS
											Peak power during Wingate test (W/kg)	NS
											Mean power during Wingate test (W/kg)	NS
											FI during Wingate test (%)	NS
							60	1300 h			Grip strength (N)	NS
											Back strength (N)	NS
											Peak power during Wingate test (W/kg)	NS
											Mean power during Wingate test (W/kg)	NS
											FI during Wingate test (%)	NS
							90	1230 h			Grip strength (N)	NS
											Back strength (N)	NS
											Peak power during Wingate test (W/kg)	NS
											Mean power during Wingate test (W/kg)	NS
											FI during Wingate test (%)	NS
Abdessalem et al. [31]	Tunisia	18 male	21 ± 3	Physically active	Individual sport (i.e., running)	≈ 7 h (self-report)	25	1300 h	215	None	HD	NS
											TD	NS
								1400 h	155		HD	↑ by 8% after nap at 1400 h vs no nap and ↑ by 6% vs nap at 1300 h
											TD	↑ by 4% after nap at 1400 h vs no nap and nap at 1300 h
								1500 h	95		HD	↑ by 7% after nap at 1500 h vs no nap and ↑ by 5% vs nap at 1300 h
											TD	↑ by 3% after nap at 1500 h vs no nap and nap at 1300 h
Boukhris et al. [32]	Tunisia	17 male	21 ± 3	Physically active	Individual sport (i.e., running)	≈ 7 h (self-report)	25	1400 h	155	Subjective sleep quality scale	HD	↑by 6% after N25 vs no nap
											TD	↑ by 3% after N25 vs no nap
											FI	NS
							35		145		HD	NS
											TD	↑ by 3% after N35 vs no nap
											FI	NS
							45		135		HD	↑ by 9% after N45 vs no nap and by 6% vs N35
											TD	↑ by 8% after N45 vs no nap, by 4% vs N35 and by 5% vs N25
											FI	NS
Daaloul et al. [41]	Tunisia	13 male	23 ± 2	Athletes	Individual sport (i.e., karate)	≈ 7 h (actigraphy)	30	1300 h	30	Actigraphy	Squat jump before the karate-specific test	NS
											Counter movement jump before the karate-specific test	NS
											Squat jump after the karate-specific test	↑
											Counter movement jump after the karate-specific test	↑
											Time to exhaustion during the karate-specific test	NS
Hsouna et al. [42]	Tunisia	20 male	21 ± 4	Physically active	Individual sport (i.e., running)	≈ 7 h (self-report)	25	1400 h	155	None	5-jump test	NS
							35	1400 h	145		5-jump test	↑ by 3.5% after N35 vs no nap
							45	1400 h	135		5-jump test	↑ by 3.7% after N45 vs no nap
Suppiah et al. [39]	Singapore	19 male	15 ± 1	Athletes	Individual sport (i.e., shooting sport)	7 h 45 min (actigraphy)	30	1430 h	45	A wireless dry electroencephalogram	Mean 2-m sprint time (s)	NS
											Mean 10-m sprint time (s)	NS
											Mean 20-m sprint time (s)	NS
											Fastest 2-m sprint time (s)	NS
											Fastest 10-m sprint time (s)	NS
											Fastest 20-m sprint time (s)	↑ by 0.76% after nap condition vs no nap
Boukhris et al. [33]	Tunisia	14 male	20 ± 3	Athletes	Team sport [i.e., soccer (*n* = 7), rugby (*n* = 3), and handball (*n* = 4)]	≈ 8–9 h (actigraphy)	40	1400 h	140	Actigraphy and subjective sleep quality scale	MVIC	↑ by 5.29% after N40 vs no nap
											HD	↑ by 7.2% after N40 vs no nap
											TD	↑ by 7.3% after N40 vs no nap
											FI	NS
							90		90		MVIC	↑ by 8.77% after N90 vs no nap and by 3.67% vs N40
											HD	↑ by 10% after N90 vs no nap
											TD	↑ by 11.3% after N90 vs no nap and by 4.3% vs N40
											FI	↓ by 33% after N90 vs no nap
Souissi et al. [36]	Tunisia	14 male	21 ± 2	Physically active	Individual sport (i.e., running)	≈ 7–8 h (actigraphy)	30	1300 h	270	None	HD	↑
											TD	↑
											FI	↓
											HD	↑
											TD	↑
											FI	↓
Boukhris et al. [34]	Tunisia	15 male	20 ± 3	Athletes	Team sport [i.e., soccer (*n* = 8), rugby (*n* = 3), and handball (*n* = 4)]	≈ 8–9 h (actigraphy)	40	1400 h	140	Actigraphy and subjective sleep quality scale	HD	↑ by 7.9% after N40 vs no nap
											TD	↑ by 7.2% after N40 vs no nap
											PD	NS
Nishida et al. [44]	Japan	11 male	21 ± 1	Athletes	Team sport (i.e., handball)	≈ 7 h (actigraphy)	20	1300 h	190	Actiheart 5	Average 20-m turnaround run (s)	NS
											Average 10-m load run (s)	NS
							60		150		Average 20-m turnaround run (s)	NS
											Average 10-m load run (s)	NS
Romdhani et al. [45]	Tunisia	13 male	20 ± 1	Athletes	Individual sport (i.e., judo)	≈ 7 h (self-report)	20	1410 h	30	None	Maximum power (Watts)	↑ after N20 vs no nap and N90
											Minimum power (Watts)	NS
											Mean power (Watts)	↑ after N20 vs no nap and N90
							90	1300 h			Maximum power (Watts)	↓ after N90 vs N20
											Minimum power (Watts)	NS
											Mean power (Watts)	↓ after N90 vs N20
Boukhris et al. [35]	Tunisia	16 male	20 ± 3	Athletes	Team sport [i.e., soccer (*n* = 8), rugby (*n* = 4), and handball (*n* = 4)]	≈ 8–9 h (actigraphy)	40	1400 h	140	Actigraphy and subjective sleep quality scale	HD	↑ after N40 vs no nap
											TD	↑ after N40 vs no nap
											FI	NS
							90		90		HD	↑ after N90 vs no nap
											TD	↑ after N90 vs no nap and N40
											FI	↓ after N90 vs no nap
Hsouna et al. [37]	Tunisia	12 male	23 ± 3	Athletes	Team sport (i.e., soccer)	≈ 6–7 h (actigraphy)	40	1400 h	140	Actigraphy	HD	↑ after N40 vs no nap
											TD	↑ after N40 vs no nap
											FI	NS
Souabni et al. [40]	France	12 male	26 ± 5	Athletes	Team sport (i.e., basketball)	≈ 7 h (actigraphy)	40	1300 h	80	Actigraphy	Best upper body power (m)	↑ after N40 vs no nap
											Mean upper body power (m)	↑ after N40 vs no nap

↑ indicates increase; ↓ indicates decrease; *HD* highest distance, *FI* fatigue index, *MVIC* maximal voluntary isometric contraction, *NM* not mentioned, *NS* not significant, *N20* 20-min nap, *N25* 25-min nap, *N35* 35-min nap, *N40* 40-min nap, *N45* 45-min nap, *N90* 90-min nap, *TD* total distance, $\dot{V}$*O*_*2max*_ maximum oxygen consumption, *PD* percentage decrement

### Quality Assessment

Of the 18 selected articles, 15 were of strong quality, and three were moderate (Table 2). Quality scores for the included studies ranged from 67.9% (moderate) to 89.3% (strong). The largest number of points were lost due to the lack of participants (94.4%) and researchers' blinding (88.9%), and the lack of control of confounding factors (44.4%) (Table 2).

**Table 2:** Quality assessment of the included studies

Study	Question described	Appropriate study design	Appropriate subject selection	Characteristics described	Random allocation	Researchers blinded	Subjects blinded	Outcome measures well defined and robust to bias	Sample size appropriate	Analytic methods well described	Estimate of variance reported	Controlled for confounding	Results reported in detail	Conclusion supported by results	Rating (%)	Study quality
Petit et al. [43]	2	2	2	2	1	0	0	2	2	2	1	2	2	2	78.6	Strong
Pelka et al. [29]	2	2	2	1	2	2	2	2	2	1	2	1	2	2	89.3	Strong
Blanchfield et al. [46]	2	2	2	2	2	0	0	1	2	2	1	1	2	2	75.0	Strong
Daaloul et al. [41]	2	1	2	2	1	0	0	2	2	2	2	2	2	2	78.6	Strong
O’Donnell et al. [30]	2	1	2	1	0	2	0	1	2	1	2	1	2	2	67.9	Moderate
Suppiah et al. [39]	2	1	2	2	1	0	0	2	2	2	2	1	2	2	75.0	Strong
Tanabe et al. [38]	1	2	2	2	0	0	0	2	1	2	2	1	2	2	67.9	Moderate
Abdessalem et al. [31]	2	2	2	1	1	0	0	2	2	2	2	1	2	2	75.0	Strong
Boukhris et al. [32]	2	2	2	2	2	0	0	2	2	2	2	1	2	2	82.1	Strong
Hsouna et al. [42]	2	2	2	1	2	0	0	2	2	2	2	1	2	2	78.6	Strong
Boukhris et al. [33]	2	2	2	2	2	0	0	2	2	2	2	1	2	2	82.1	Strong
Souissi et al. [36]	2	2	2	2	2	0	0	2	2	2	2	1	2	2	82.1	Strong
Boukhris et al. [34]	2	2	2	2	2	0	0	2	2	2	2	1	2	2	82.1	Strong
Nishida et al. [44]	2	2	2	2	2	0	0	2	1	2	2	1	2	2	78.6	Strong
Romdhani et al. [45]	2	2	2	2	2	0	0	2	2	2	2	1	2	2	82.1	Strong
Boukhris et al. [35]	2	2	2	2	2	0	0	2	2	2	2	1	2	2	82.1	Strong
Hsouna et al. [37]	2	2	2	2	2	0	0	2	2	2	2	1	2	2	82.1	Strong
Souabni et al. [40]	2	2	2	2	2	0	0	2	2	2	2	1	2	2	82.1	Strong
% of lost points (%)	2.8	8.3	0.0	11.1	22.2	88.9	94.4	5.6	5.6	5.6	5.6	44.4	0.0	0.0		

Yes = 2, partial = 1, no = 0

### Impacts of Daytime Napping Following Normal Sleep on Physical Performance

#### Impacts of Napping on 5-m Shuttle Run Test

##### Highest Distance (HD)

**Fig. 2:**
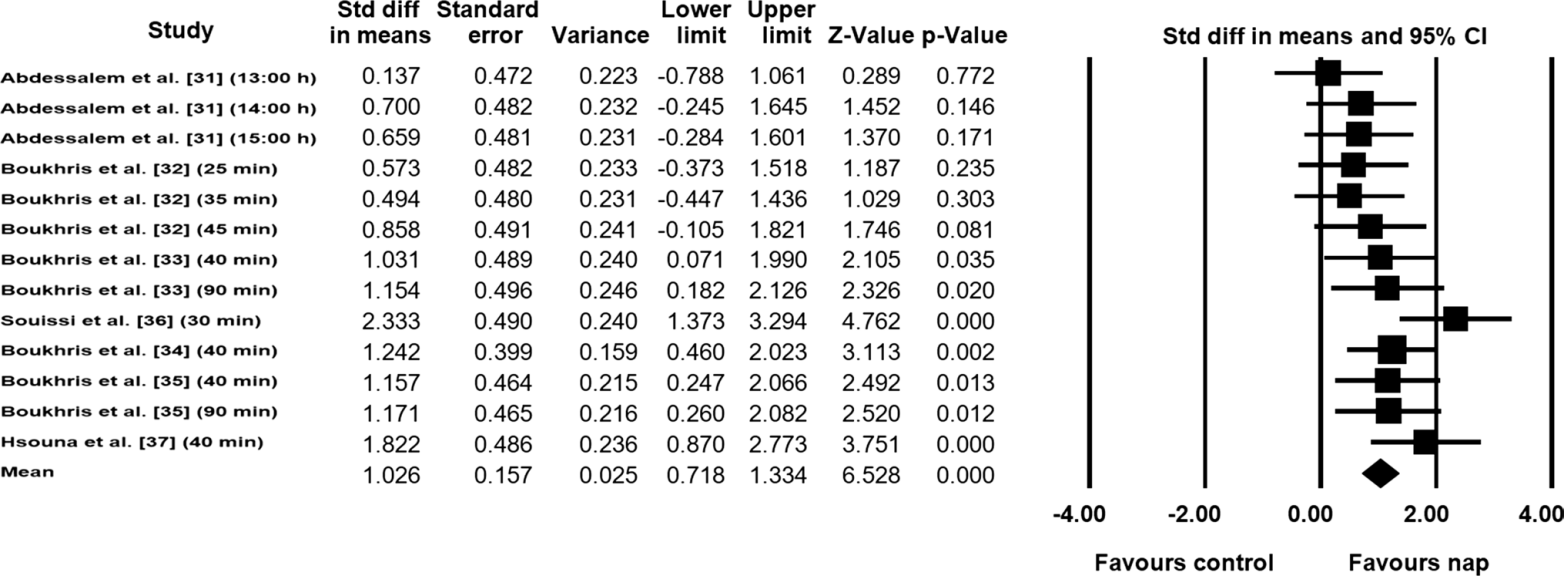
Forest plot for the impact of daytime napping following normal sleep on highest distance during the 5-m shuttle run test. *Std diff* standard difference, *CI* confidence intervals

**Fig. 3:**
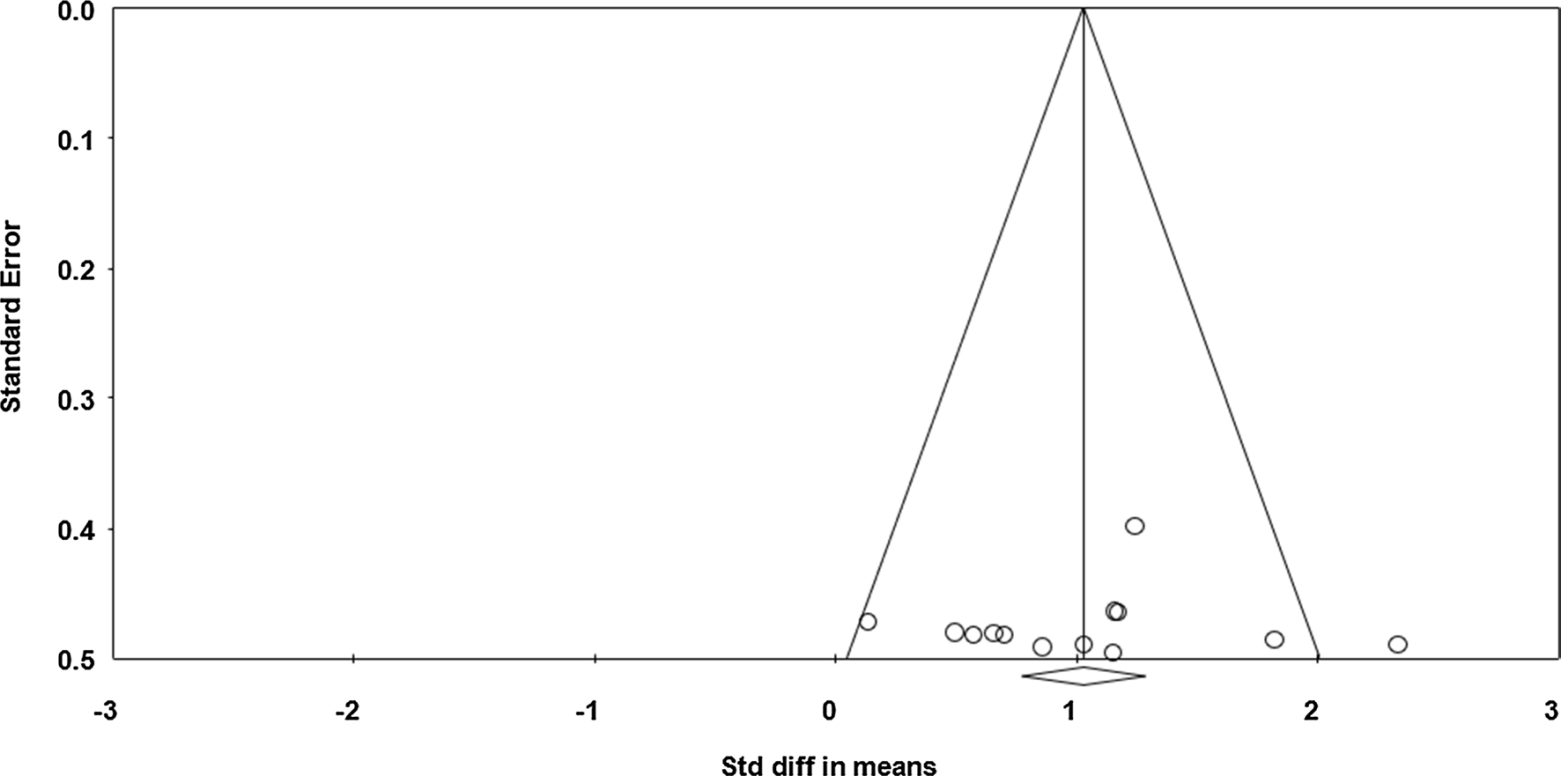
Funnel plot for highest distance during the 5-m shuttle run test showing no evidence of publication bias. *Std diff* standard difference

Data from seven studies (*n* = 106 athletes), including 13 comparisons (no-nap vs nap), were pooled in the meta-analysis [31–37]. Pooling findings yielded a significant positive moderate ES of 1.026 (standard error [SE] 0.157; 95% CI 0.718–1.334; *Z* value = 6.528; *p* < 0.001; Fig. 2), with non-significant heterogeneity (*Q* = 17.157; *df* = 12; *p* = 0.144; *I*^2^ = 30.1%). The ES was translated to a difference in means of 8.2 m (95% CI 5.4–11.1). Visual inspection of the funnel plot (Fig. 3) showed no evidence of publication bias, a conclusion confirmed by Begg and Mazumdar’s rank correlation test and by Egger’s linear regression test (Table 3). Duval and Tweedie’s trim-and-fill test identified two studies to trim and a ‘true ES’ of 1.155. Both sensitivity analysis and cumulative meta-analysis confirmed the reliability and stability of the current findings (Figs. S1, S2, see ESM).

**Table 3:** Results of the Begg and Mazumdar’s rank correlation test and Egger’s linear regression test

	Begg and Mazumdar’s rank correlation test							Egger’s linear regression test					
	Kendall’s *S* statistic P–Q	*τ* without continuity correction	*z*	*p*	*τ* with continuity correction	*z*	*p*	Intercept	SE	95% confidence interval	*t*	*df*	*p*
Highest distance	10.00	0.12	0.61	0.27	0.11	0.54	0.29	− 0.76	6.01	− 13.99 to 12.46	0.12	11	0.45
Total distance	14.00	0.17	0.85	0.19	0.16	0.79	0.21	0.92	3.19	− 6.10 to 7.95	0.28	11	0.38
Fatigue index	29.00	0.64	2.59	0.004	0.62	2.5	0.006	11.04	3.16	3.75 to 18.34	3.49	8	0.004
Muscle force	− 5.00	− 0.09	0.38	0.34	− 0.07	0.31	0.37	− 8.03	2.12	− 12.84 to − 3.23	3.78	9	0.002

*df* degrees of freedom, *SE* standard error

##### Total Distance (TD)

**Fig. 4:**
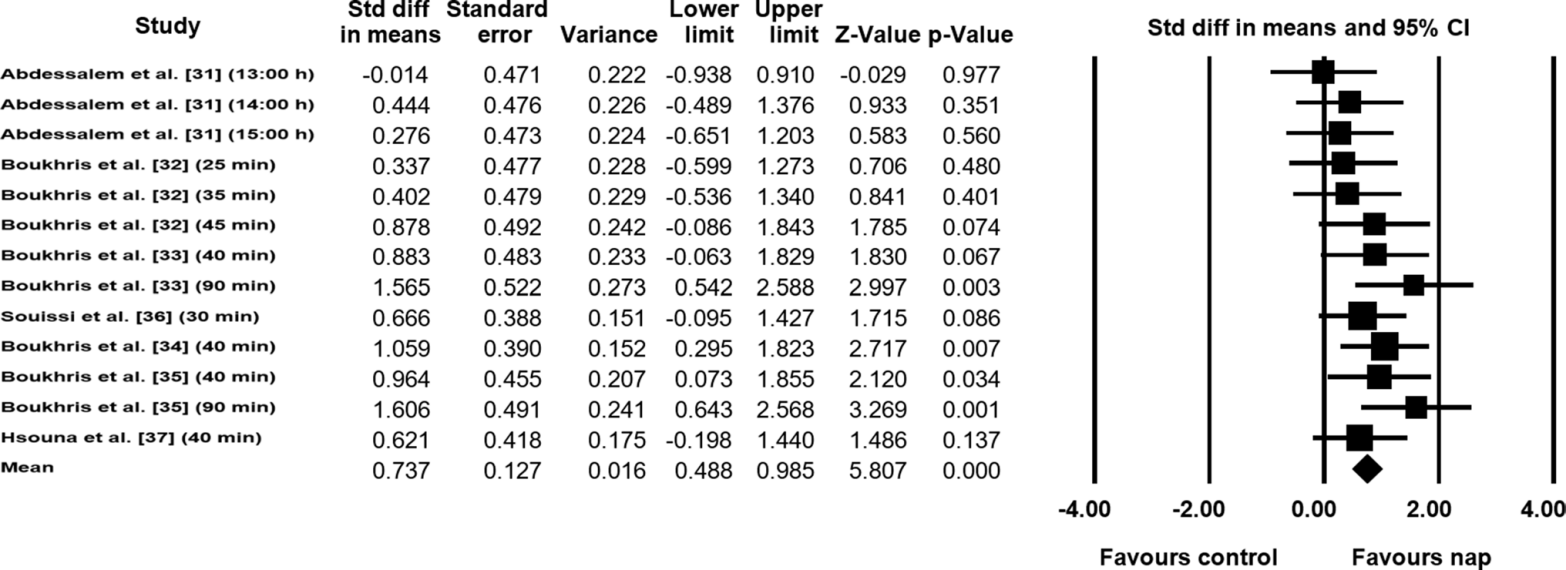
Forest plot for the impact of daytime napping following normal sleep on total distance during the 5-m shuttle run test. *CI* confidence intervals, *Std diff* standard difference

**Fig. 5:**
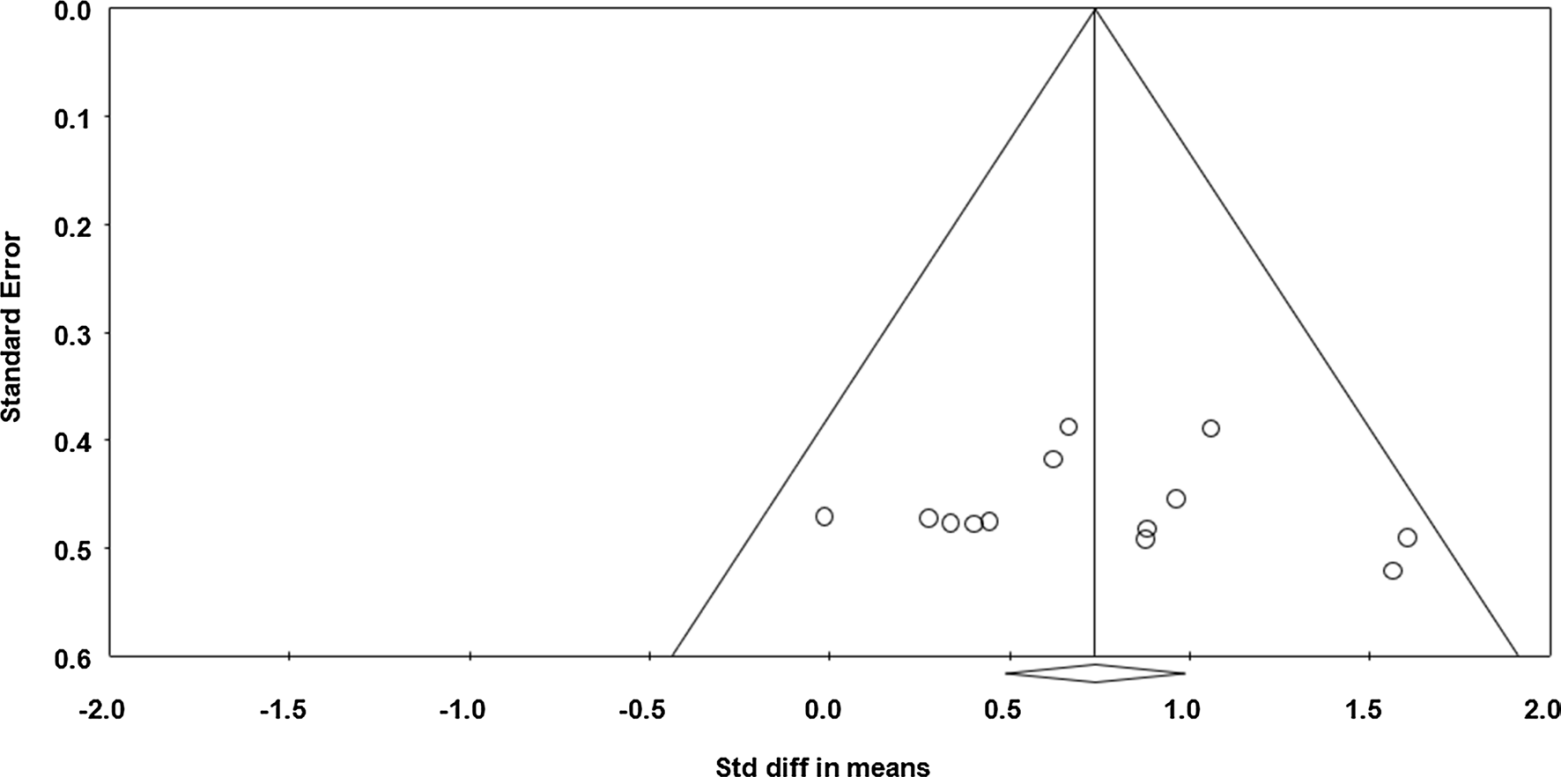
Funnel plot for total distance during the 5-m shuttle run test showing no evidence of publication bias. *Std diff* standard difference

Data from seven studies (*n* = 106 athletes), including 13 comparisons (no-nap vs nap), were pooled in the meta-analysis [31–37]. Pooling findings yielded a significant positive moderate ES of 0.737 (SE 0.127; 95% CI 0.488–0.985; *Z* value = 5.807; *p* < 0.001; Fig. 4), with non-significant heterogeneity (*Q* = 11.916; *df* = 12; *p* = 0.452; *I*^2^ = 0%). The ES was translated to a difference in means of 38.5 m (95% CI 18.4–58.6). Visual inspection of the funnel plot (Fig. 5) showed no evidence of publication bias, a conclusion confirmed by Begg and Mazumdar’s rank correlation test and by Egger’s linear regression test (Table 3). Duval and Tweedie’s trim-and-fill test did not identify any missing studies. Both sensitivity analysis and cumulative meta-analysis confirmed the reliability and stability of the current findings (Figs. S3, S4, see ESM).

##### Fatigue Index (FI)

**Fig. 6:**
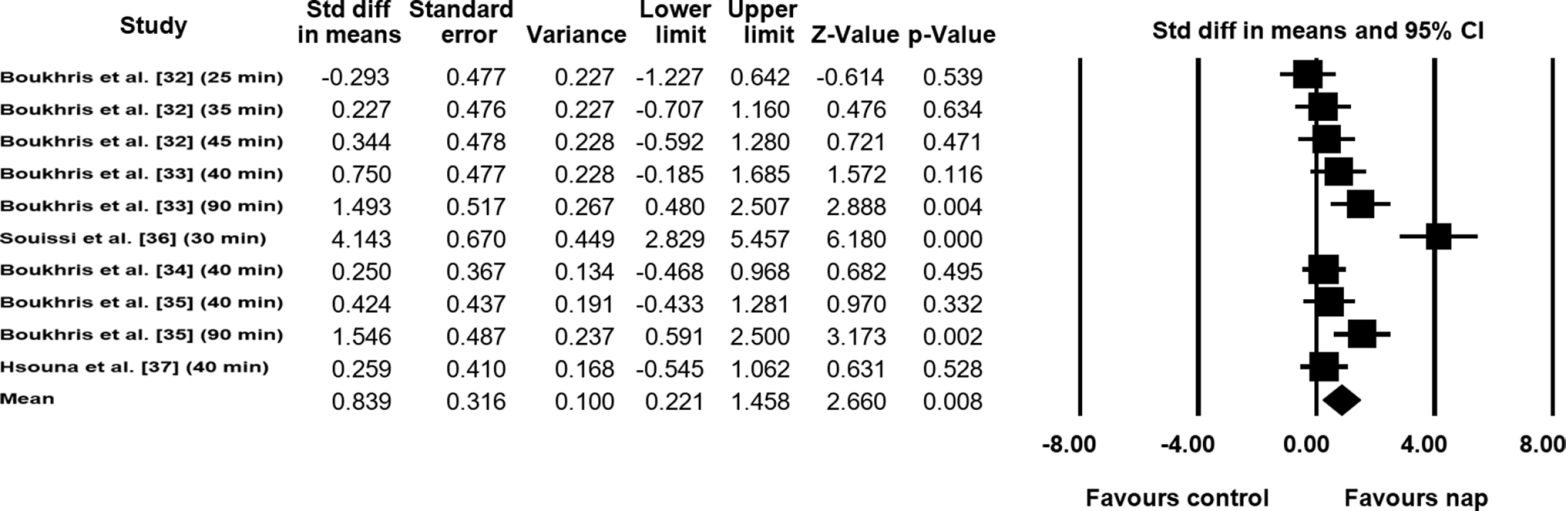
Forest plot for the impact of daytime napping following normal sleep on fatigue index during the 5-m shuttle run test. *CI* confidence intervals, *Std diff* standard difference

**Fig. 7:**
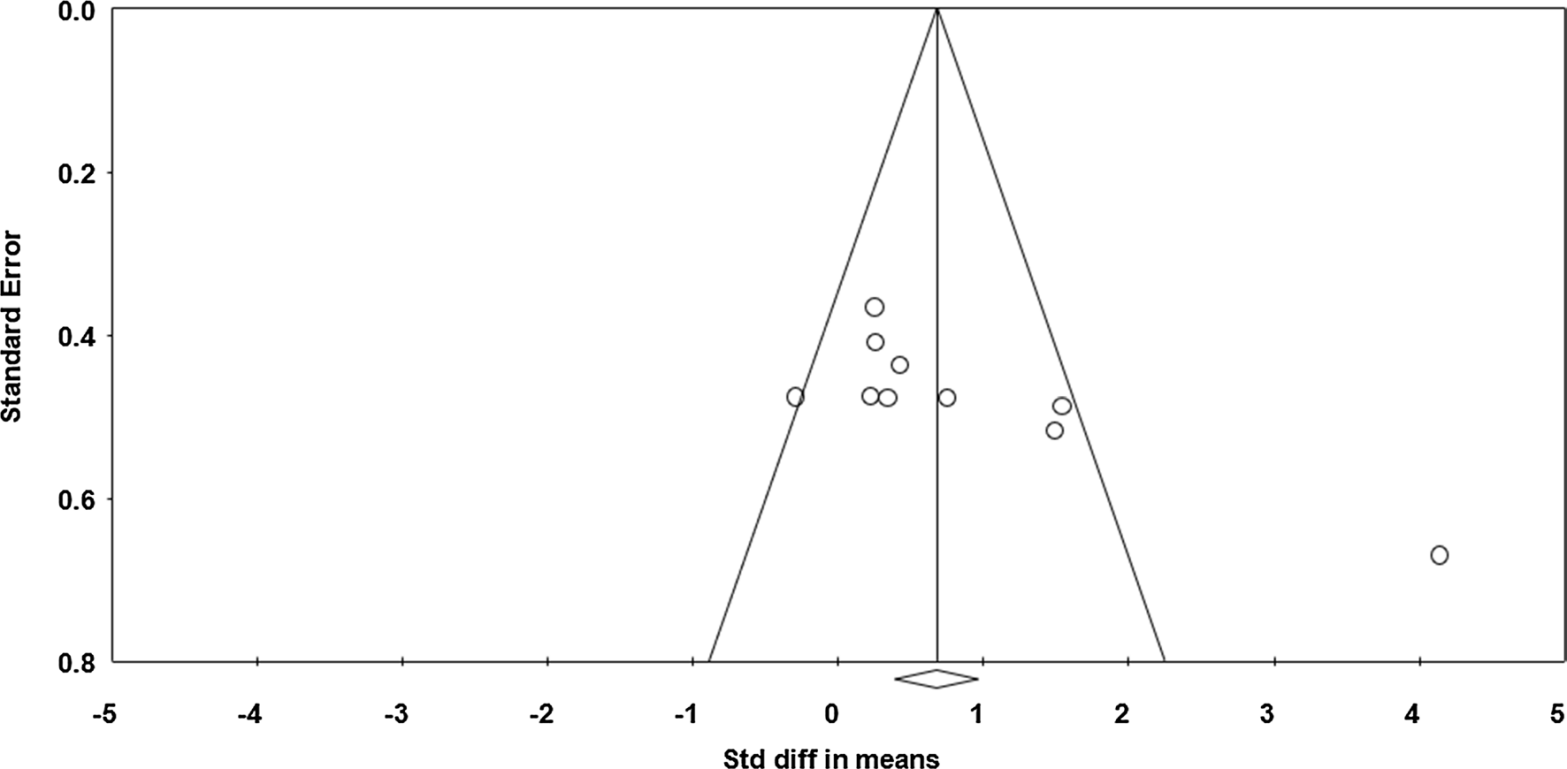
Funnel plot for fatigue index during the 5-m shuttle run test showing evidence of publication bias. *Std diff* standard difference

Data from six studies (*n* = 88 athletes), including 10 comparisons (no-nap vs nap), were pooled in the meta-analysis [32–37]. Pooling findings yielded a significant, positive, moderate ES of 0.839 (SE 0.316; 95% CI 0.211–1.458; *Z* value = 2.660; *p* = 0.008; Fig. 6), with significant heterogeneity (*Q* = 40.679; *df* = 9; *p* < 0.001; *I*^2^ = 77.9%). The ES was translated to a difference in means of 2.5% (95% CI 1.6–3.5). Visual inspection of the funnel plot (Fig. 7) showed evidence of publication bias, a conclusion confirmed by Begg and Mazumdar’s rank correlation test and by Egger’s linear regression test (Table 3). However, Duval and Tweedie’s trim-and-fill test did not identify any missing study. Both sensitivity analysis and cumulative meta-analysis confirmed the reliability and stability of the current findings (Figs. S5, S6, see ESM).

Meta-regressions showed no impact of age (coefficient = − 0.38; SE 0.35; 95% CI − 1.07 to 0.30; *Z* value = − 1.10; *p* = 0.27), level of practice (*Q* = 0.09; *df* = 1; *p* = 0.76), activity (*Q* = 0.09; *df* = 1; *p* = 0.76), and nap duration (coefficient = 0.01; SE = 0.01; 95% CI − 0.01 to 0.04, *Z* value = 0.86; *p* = 0.39). However, meta-regressions showed an impact of time between the end of napping and the exercise (coefficient = 0.01; SE = 0.007; 95% CI 0.0003–0.027; *Z* = 2.0; *p* = 0.04).

#### Impacts of Napping on Muscle Force

**Fig. 8:**
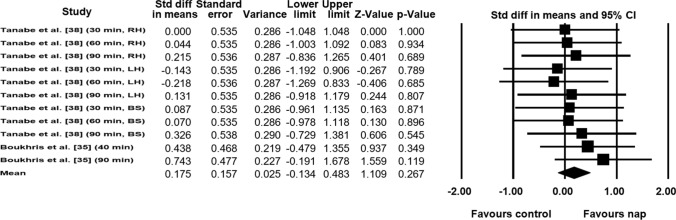
Forest plot for the impact of daytime napping following normal sleep on muscle force. *Std diff* standard difference, *CI* confidence intervals, *RH* right hand, *LH* left hand, *BS* back strength

**Fig. 9:**
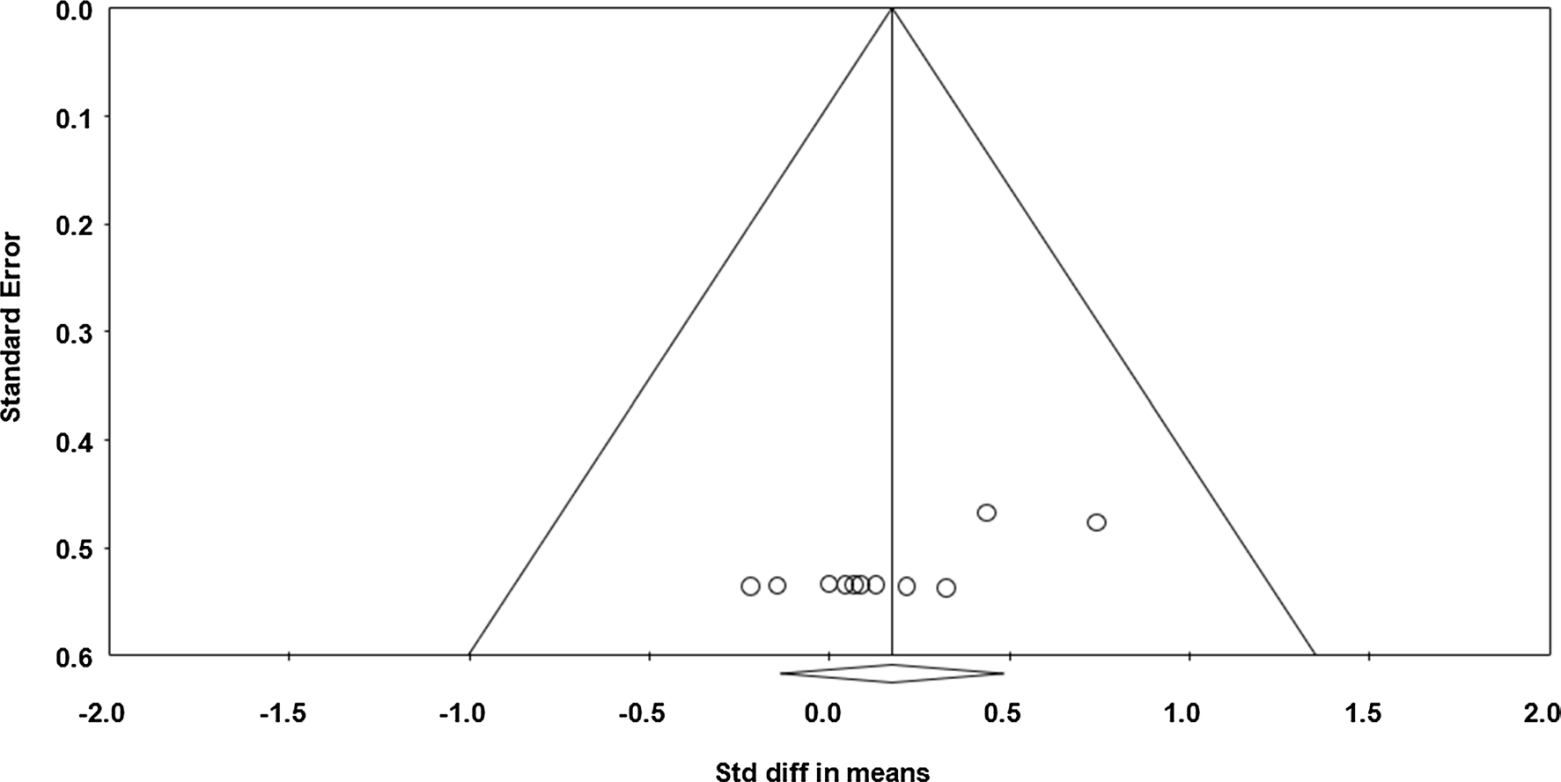
Funnel plot for muscle force showing evidence of publication bias. *Std diff* standard difference

Data from two studies (*n* = 21 athletes), including 11 comparisons (no-nap vs nap), were pooled in the meta-analysis [33, 38]. Pooling findings yielded a non-significant, positive, and small ES of 0.175 (SE 0.157; 95% CI − 0.134 to 0.483; *Z* value = 1.109; *p* = 0.267; Fig. 8), with non-significant heterogeneity (*Q* = 2.95; *df* = 10; *p* = 0.98; *I*^2^ = 0%). Visual inspection of the funnel plot (Fig. 9) showed evidence of publication bias, a conclusion confirmed by Egger’s linear regression test (Table 3). However, Begg and Mazumdar’s rank correlation test showed no evidence of publication bias (Table 3). Duval and Tweedie’s trim-and-fill test identified three studies to trim and a ‘true ES’ of 0.27 was calculated. Both sensitivity analysis and cumulative meta-analysis confirmed the reliability and stability of the current findings (Figs. S7, S8, see ESM).

#### Impacts of Napping on Sprint Performance

One study examined the impact of napping following normal sleep on sprint performance [39]. The authors failed to observe any significant positive effect of a 30-min nap on mean 2-m sprint, mean 10-m sprint, mean 20-m sprint, fastest 2-m sprint, and fastest 10-m sprint. However, for the fastest 20-m sprint, a significant increase in 20-m sprint time was observed after napping compared with the no-nap condition. Specifically, the mean 20-m sprint time increased from 3.385 s in the no-nap condition to 3.411 s after the nap condition.

#### Impacts of Napping on Power Performance

Only one study measured best and mean upper body power [40] after a 40-min nap, and both were increased significantly, by 6.8% and 5.8% respectively, compared with the no-nap condition.

#### Impacts of Napping on Jump Performance

In relation to jumping performance, three studies examined the impact of napping following normal sleep on jump performance [30, 41, 42]. It was reported that short naps (i.e., < 20 min) enhanced the peak jump velocity, without any significant effect on mean jump velocity and height jump [30]. However, long naps (i.e., ≥ 20 min) enhanced the mean jump velocity, without any significant effect on peak jump velocity and height jump [30]. Moreover, Hsouna et al. [42] reported that the 5-jump test performance was improved following a 35-min and a 45-min nap in comparison with a no-nap condition. However, Daaloul et al. [41] observed no significant effects of a 30-min nap on squat jump and counter movement jump performance before an exhaustive task (i.e., the karate-specific test). In contrast, squat jump and countermovement jump performance recorded after an exhaustive task were improved after napping compared with a no-nap condition. Specifically, the difference between the squat jump performances pre to post the exhaustive task was − 0.8 ± 2.3 cm in the nap condition compared with − 2.1 ± 3.8 cm in the no-nap condition. The difference in countermovement jump performance pre to post the exhaustive task was − 3.1 ± 1.3 cm in the nap condition compared with − 3.6 ± 1.7 cm in the no-nap condition.

#### Impacts of Napping on 30-Second Wingate Test

For the 30-s Wingate test, two studies failed to observe any significant effect of napping (i.e., 20 min [43], 30 min, 60 min, and 90 min [38]).

#### Impacts of Napping on Repeated Sprint

Three studies examined the impact of napping following normal sleep on repeated sprint performance [29, 44, 45]. Pelka et al. [29] reported that a 25-min nap increased the average maximum speed of the 6 × 4 s sprints. Conversely, Romdhani et al. [45] showed a significant increase in maximum speed and power after a 20-min nap, without any improvement after a 90-min nap. However, Nishida et al. [44] failed to observe any significant effect of napping (i.e., 20-min and 60-min naps) in a repeated sequential trial consisting of a 20-m consecutive turnaround run, and 10-m run with a load.

#### Impacts of Napping on Endurance Performance

Two studies examined the impact of napping following normal sleep on endurance performance [41, 46]. Blanchfield et al. [46] reported that running time to exhaustion at 90% maximum oxygen consumption ($\dot{V}$O_2max_) was not improved after napping compared with a no-nap condition for the whole group tested. However, the authors found that running time to exhaustion was improved for only five runners who had lower duration of sleep than the remaining participants (382 ± 39 min vs 449 ± 24 min) the previous night. However, Daaloul et al. [41] failed to observe any significant effect of napping (i.e., 30-min nap) in time to exhaustion during the Karate-specific test.

## Discussion

This is the first meta-analysis evaluating the effects of daytime napping, following normal night-time sleep, on athletic performance. The present findings showed that diurnal napping enhanced physical performance during the 5-m shuttle run test, but did not enhance measures of muscle force. However, the low number of studies on sprint performance, jump performance, Wingate test, repeated sprint, and endurance performance precluded drawing firm conclusions on these outcomes. Our results are in agreement with the previous reviews (i.e., two systematic reviews [13, 14] and one narrative review [12]) that support the use of daytime napping to enhance athletic performance.

### Effect of Daytime Napping on 5-m Shuttle Run Test (5MSRT)

The results of the current meta-analysis demonstrated that daytime napping following a full night of sleep improved physical performance (i.e., HD, TD, and FI) during the 5MSRT in athletes and physically active men. The meta-analytic pooling of HD data showed that HD increased by 8.2 m in favor of napping compared with the no-nap condition. Many physiological elements are part of the measure of HD, particularly agility, speed, and alactic or adenosine triphosphate and phosphocreatine (ATP-PCr) capacity [31]. During the first 30-s shuttle (i.e., HD), almost all the creatine phosphate store is utilized, and the capacity to do muscle work is associated with the ability to utilize the high-energy phosphate pool [47]. Indeed, the participant could generate more mechanical work and run faster if there is greater assistance of more chemical energy from the high-energy phosphate pool [47], which could potentially be due to napping.

In addition, the meta-analytic pooling of TD data showed that TD increased by 38.5 m in favor of napping compared with the no-nap condition. Therefore, daytime napping appears to have a positive influence on anaerobic capacity and metabolic recovery, as TD covered during the six 30-s shuttle runs is an indicator of anaerobic capacity and the ability to recover rapidly between sprints.

Furthermore, the meta-analytic pooling of FI data showed that FI increased by 2.5% in favor of napping compared with the no-nap condition, which also reflects the positive impact of napping on the ability to recover quickly between sprints during the 5MSRT. For FI, the meta-regression conducted in the current study indicates that FI during the 5MSRT may be influenced by the time between the end of napping and the exercise, indicating that a longer duration between the end of napping and the exercise’s start time may generate a larger decrease in FI. This suggests that sufficient time should be provided to athletes’ post-nap to avoid the negative effect of sleep inertia that appears immediately after waking from sleep. Indeed, sleep inertia is defined as “the transitional state between sleep and wakefulness characterized by a short-term decrease in arousal and performance” [48]. In addition, it was reported that fatigue perception increased after waking, apparently due to sleep inertia [49]. Therefore, to recover quickly between sprints during the 5MSRT and achieve lower FI, athletes should allow sufficient time before beginning exercise for the purpose of avoiding the negative effects of sleep inertia. It is worth noting that meta-regression is ineffective for demonstrating a cause-and-effect relationship, and therefore, the results should be interpreted with caution [50]. Additionally, more research is required to specify the exact duration needed to avoid these effects following naps of varying durations.

### Effect of Daytime Napping on Muscle Force

The current meta-analysis showed that muscle force remained unchanged after napping compared with the no-nap condition. A possible explanation for the absence of significant improvement of muscle force following a diurnal nap is the difference between the mode of exercise tested in the included studies. For example, Tanabe et al. [38] tested the grip strength of the right and left hand, and back strength, following three nap durations (i.e., 30 min, 60 min, and 90 min), and did not find any significant impact of any nap durations. However, Boukhris et al. [33] tested maximal voluntary isometric contraction of the right leg following two nap durations (i.e., 40 min and 90 min), and found significant improvements after both nap durations with a greater improvement after the 90-min nap. Another possible explanation for the absence of significant improvement in muscle force is the difference between the time of day of napping in the included studies. Indeed, 12:30 h, 13:00 h, and 13:30 h were the times of napping for the three durations (i.e., 30 min, 60 min, and 90 min, respectively) in the study of Tanabe et al. [38], while 1400 h was the time of napping for the two durations (i.e., 40 min and 90 min) in the study of Boukhris et al. [33]. In this context, it was reported that napping following normal sleep at 14:00 h and 15:00 h produced a significant enhancement of physical performance, whereas napping at 13:00 h did not influence physical performance [31]. Therefore, a nap taken between 14:00 h and 15:00 h could be more beneficial than earlier naps, especially following normal sleep. In this context, Lastella et al. [13] suggested that between 13:00 h and 16:00 h is the optimal time to nap due to the high level of sleepiness that occurs during that period. However, Lastella et al. [13] did not mention if this period was better suited to individuals who slept normally or experienced sleep restriction. Accordingly, Abdessalem et al. [31] suggested taking an earlier nap following sleep restriction, because of the high level of fatigue faced, and later naps following normal sleep. Although this suggestion seems feasible, future studies are required to determine the best time of day for napping following normal or restricted sleep. Future studies are also required to clarify the effect of napping following normal sleep on muscle force.

### Effect of Daytime Napping on Performance During Short-Term Maximal Exercise

Relatively few studies have investigated the effect of daytime napping following normal sleep on performance during short-term maximal exercise. For example, only one study examined the effect of a 30-min nap following normal sleep on sprint performance, and no improvement was detected [39]. Suppiah et al. [39] suggested that sleep inertia was responsible for the absence of physical performance enhancement after napping. In reality, in order to avoid the negative effect of sleep inertia, around 1 h after waking should be allowed for athletes before exercise [33], which was not the case in the study by Suppiah et al. [39], who allowed 45 min before exercise. However, regarding jump performance, three studies investigated the effect of napping following normal sleep, and the results were conflicting. Indeed, no improvements were detected in squat jump and countermovement jump after a 30-min nap in the study by Daaloul et al. [41], while significant improvements in peak and mean jump velocity during countermovement jump were detected in the study by O’Donnell et al. [30]. In addition, significant improvement in the 5-jump test was observed in the study by Hsouna et al. [42]. The contrasting results could be related to the differences in study design (i.e., tests used, nap durations, and time to avoid sleep inertia).

### Effect of Daytime Napping on Performance During the 30-s Wingate Test and Repeated Short-Term Maximal Exercises

Only two studies investigated the effect of daytime napping following normal sleep on performance during the 30-s Wingate test [38, 43], and they failed to observe any improvements after napping. However, three studies investigated the effect of daytime napping following normal sleep on performance during repeated sprints, and the results were in favor of napping [29, 44, 45]. The contrasting results could be related to the type of exercise. Indeed, the 30-s Wingate test is a different testing modality than the other sprint exercises used [14]. Repeated sprint exercises may recruit more muscle mass compared with cycle ergometer exercise during the Wingate test, and the repetition of maximal effort in repeated sprint exercises results in a high accumulation of lactate as opposed to a one-off performance [14]. Accordingly, it could be that napping has a powerful effect on highly fatiguing exercises. The longer and more intense the exercise, the more recovery is needed before the exercise to increase energy stock, which could explain the significant effect of napping on repeated sprint exercises more than other maximal short-term exercises. Nevertheless, future studies are required to clarify the effect of napping following normal sleep on performance during the 30-s Wingate test and repeated short-term maximal exercises, as current studies are limited in number.

### Effect of Daytime Napping on Endurance Performance

Only two studies investigated the effect of daytime napping following normal sleep on endurance performance [41, 46], and the results were conflicting. In fact, no improvements were detected after a 30-min nap in the study by Daaloul et al. [41], while significant improvements were reported after a 40-min nap in the study by Blanchfield et al. [46]. These contrasting results could be related to sleep inertia. Indeed, only 30 min was allowed for participants in the study by﻿ Daaloul et al. [41] to avoid sleep inertia, which may not be sufficient. In addition, a nap duration of 30 min could be too short to observe any physical performance improvement. Nevertheless, future studies are required to clarify the effect of different nap durations following normal sleep on endurance performance, as current studies are limited in number.

### Potential Mechanisms Underlying the Beneficial Effect of Daytime Napping on Physical Performance

Thirteen studies [29–37, 40–42, 45] included in the current review showed an improvement in physical performance after napping; however, five studies [38, 39, 43, 44, 46] failed to observe an improvement in physical performance following a nap. These contradictions could be related to the duration of napping used in each study [33]. Indeed, it has been reported that the duration of a nap influenced its efficacy in enhancing physical performance [32]. More importantly, the beneficial effect shown after napping could be related to perceptual/psychological and physiological aspects of napping [12, 33, 35, 46]. In this context, a nap could enhance physical performance as it can significantly increase alertness [33, 36] and decrease sleepiness [33–35]. As physical performance is related to alertness level, a diurnal nap could decrease the level of sleepiness [33–35], allowing athletes to feel more alert, which in turn may be responsible for enhancing physical performance [33, 36]. Moreover, it has been revealed that physical performance and mood states have a direct relationship with sleep quantity and quality [51, 52]. Therefore, the improvement of mood states shown after napping could help individuals to reach peak physical performance. Additionally, taking a nap could help lower feelings of stress by allowing a brief period of calmness and relaxation [42]. During this period, physiological and psychological systems undergo a restorative process, which can lower the body's levels of stress hormones (e.g., cortisol and epinephrine) [53]. Additionally, taking a nap could give athletes a mental break from the stresses of training and competition. Furthermore, some studies reported that daytime napping has a positive impact on sports performance by reducing the sense of effort (i.e., rating of perceived exertion) [33–35, 46]. In addition, the amount of slow-wave sleep during napping could also explain the improvement in physical performance [32, 33]. Indeed, slow-wave sleep is imperative for good recuperation, aids in the restoration of physical damage, and lowers anxiety and stress [32, 33]. Slow-wave sleep episodes during napping could potentially ease peripheral and neural cellular restoration, and have a role in energy conservation, most apparently because of higher parasympathetic activation [12]. Furthermore, it seems that the higher the proportion of slow-wave sleep contained in the nap, the greater the benefits for athletic performance [12]. The amount of time spent in slow-wave sleep increases continuously with increasing nap duration [12]. In this context, Tanabe et al. [38] reported that 1.4 min, 13.7 min, and 16.0 min of slow-wave sleep are observed in 30 min, 60 min, and 90 min naps, respectively. Moreover, when rapid eye movement (REM) sleep is observed during a nap, muscle contraction efficiency might be improved, with greater enhancement of athletic performance [54]. However, future studies examining naps including REM sleep or with a full cycle of sleep are required. In addition, it has been shown that a diurnal nap is considered an efficient method of minimizing the increase in muscle damage and inflammation during repeated maximal running sprints [34]. Therefore, beginning each physical exercise with lower muscle damage and inflammation due to the impact of napping could potentially slow down the onset of fatigue and, consequently, lead to enhanced performance.

Another plausible reason for the benefits following napping might relate to cardiac function. Sleep is profoundly responsible for cardiovascular regulation, and the connection between sleep and the cardiovascular system has to be considered bidirectional [55]. There is an augmentation in the parasympathetic effect on the heart during the switch from wake to non-REM sleep [35]. An increase in parasympathetic activity in response to napping contributes to enhanced physical performance, and longer daytime naps were more effective in the study by Boukhris et al. [35]. In this context, Boukhris et al. [35] illustrated that a 90-min nap resulted in a greater influence on parasympathetic activity in comparison with a 40-min nap, potentially due to the fact that a 90-min nap opportunity could contain all stages of sleep. Indeed, higher parasympathetic activity during napping is evidenced by a decline in heart rate and a rise in heart rate variability [35], which is used as an indicator of the recovery state. Boukhris et al. [35] explained that the enhancement of physical performance after napping is related to reducing sympathetic hyperactivity and pro-inflammatory cytokines, which are the result of increased parasympathetic activity. This indicates that daytime napping could work as a ‘mini-cardiovascular’ break [35], and, as a result, napping would engender greater recovery, which is crucial for an athlete's performance.

### Methodological Considerations When Implementing Napping

Objective measurement of sleep during napping was the main limitation of the majority of studies in the current review. Only two studies used polysomnography (i.e., the gold standard of sleep measurement) [38, 43]. In contrast, six studies did not use any objective tool to measure sleep during napping; instead, they only provided an estimate of nap duration [29–31, 36, 42, 45]. Other studies opted to measure napping using subjective measures [32], Actiheart [44], and a wireless dry electroencephalogram [39]. Seven studies measured sleep during napping using actigraphy [33–35, 37, 40, 41, 46]. All the methods, other than polysomnography, used to measure sleep during napping did not give information about sleep stages, which is crucial for understanding the underlying mechanisms of the benefits of napping. Therefore, using an objective measurement for sleep during napping, which differentiates the sleep stages, is required for future studies. Nevertheless, participants' sleep during napping could be affected by polysomnography equipment. Hence, technological advancements (e.g., Somfit or Dreem [56]) that can accurately measure electroencephalogram (EEG), electrocardiogram (ECG), electrooculogram (EOG) and other signals similar to polysomnography, but that are less intrusive and can be utilized at home, will make it much easier to evaluate sleep staging during naps.

Sleep inertia is one factor that should be taken into consideration when implementing a diurnal nap. Unfortunately, no studies confirmed the exact time needed to avoid the negative effect of sleep inertia for athletes. However, for non-athletes (i.e., inactive individuals), it was reported that performances could be impaired for up to 2 h post-wake [13]. The time allowed for participants to overcome sleep inertia in the included studies varied from 30 to 270 min [30–45]. Accordingly, future research should examine the effect of sleep inertia following daytime napping on athletic performance, focusing on which strategies should be added to minimize the effect of sleep inertia.

A diurnal nap could perturb the following night's sleep, especially sleep onset latency, which is another factor that should be taken into consideration. A number of factors could be responsible, such as prior sleep debt, nap duration, and time of day of napping [13]. Petit et al. [43] reported that after a 20-min nap, there was an increase in sleep onset latency. Although there is currently insufficient evidence to support a causal relationship between daytime napping and reduced quality or quantity of nighttime sleep in the general population [57, 58], certain studies have reported potential negative effects. For example, Campbell et al. [59] reported that it took older healthy men and women who had taken a nap 6.3 min longer to fall asleep compared with those who had not taken a nap. As a consequence, future studies should examine if daytime napping will affect the following night's sleep, and if so, which strategies could be implemented in order to avoid this disruption.

### Strengths and Weaknesses

This is the first systematic review and meta-analysis on the impacts of daytime napping following normal night-time sleep on physical performance in physically active individuals and athletes. The strengths of the current study are the comprehensive coverage of the available literature and a careful appraisal of its quality. Moreover, seven databases were searched without time limitations and studies published in all languages were included. The paucity of studies that objectively assessed nap and nocturnal sleep durations is a limitation. Another limitation is that the results may not be generalizable to the broader population, as only physically active individuals and athletes were included as participants. All studies that implemented napping following sleep restriction or deprivation were excluded in the present meta-analysis due to the limited number of studies, and therefore, analysis of those studies is beyond the scope of this paper. Moreover, meta-analytical calculations were necessarily limited to 5MSRT (i.e., HD, TD, and FI) and muscle force due to the limited number of studies and diverse methodology and outcome data for other aspects of physical performance. Only two studies among the 18 included studies used the gold standard of sleep measurement (i.e., polysomnography). Therefore, further studies, preferably based on objective sleep measures that provide information about sleep stages, are warranted. Moreover, it is worth noting that none of the studies included in this review assessed participants' sleep habits over a prolonged period prior to testing. Therefore, future studies should incorporate a minimum 1-week assessment of participants' sleep to ensure that chronic sleep deprivation is not a confounding factor.

## Conclusion

Napping from 25 to 90 min, following normal night-time sleep, increases physical performance during the 5-m shuttle run test in physically active individuals and athletes. On the other hand, the present meta-analysis does not demonstrate that a diurnal nap could improve muscle force. No firm conclusions can be drawn regarding the impacts of napping on other physical performance (e.g., sprint, jump, power, 30-s Wingate, and endurance performance) due to the limited number of available studies. Our meta-regression analysis revealed that moderator variables such as population size, age, level of practice, activity, time of day of napping, and nap duration may not influence the effects of napping on highest distance and total distance during the 5-m shuttle run test, nor muscle force during grip strength and maximal voluntary isometric contraction. However, fatigue index may be influenced by the time between the end of napping and the exercise.

### Supplementary Information

Below is the link to the electronic supplementary material.

Supplementary file1 (DOCX 450 KB)

## Declarations

### Data Availability

All data and materials are available within the article or supplementary materials.
