# Supplementary material for: The Impact of Daytime Napping Following Normal Night-Time Sleep on Physical Performance: A Systematic Review, Meta-analysis and Meta-regression
Source: Sports Med. 2023 Sep 12;54(2):323–45. doi: 10.1007/s40279-023-01920-2 (PMC10933197; doi:10.1007/s40279-023-01920-2)
Supplement: Supplementary file 1 — Supplementary file1 (DOCX 450 KB) [file 40279_2023_1920_MOESM1_ESM.docx]

**Table S1.** Search strategy.

| **Pubmed 30/08/2022** | | |
| --- | --- | --- |
| [1] | nap[tw] OR napping[tw] OR "daytime nap*"[tw] OR "daytime sleep*"[tw] OR siesta[tw] | **[1] AND [2] AND [3]**  **N=252** |
|  | **N=15,775** |  |
| [2] | "physically active*"[tw] OR "physical activity*"[tw] OR athletes*[tw] |  |
|  | **N=198,952** |  |
| [3] | "Sports"[Mesh] OR sport*[tw] OR performance*[tw] OR "Athletic Performance"[Mesh] OR "athletic performance*"[tw] OR "Physical Functional Performance"[Mesh] OR "physical performance*"[tw] OR "jump performance*"[tw] OR "repeated sprint*"[tw] OR sprint*[tw] OR "sprint performance*"[tw] OR speed*[tw] OR "Muscle Strength"[Mesh] OR strength*[tw] OR "anaerobic performance*"[tw] OR "aerobic performance*"[tw] OR power*[tw] OR "Physical Endurance"[Mesh] OR endurance*[tw] OR “Exercise” [Mesh] OR exercise*[tw] OR "high-intensity exercise*"[tw] OR "repeated high-intensity exercise*"[tw] |  |
|  | **N=2,893,130** |  |
| **Web of science 30/08/2022** | | |
| [1] | nap OR napping OR "daytime nap" OR "daytime sleep" OR siesta | **[1] AND [2] AND [3]**  **N=216** |
|  | **N=20,818** |  |
| [2] | "physically active" OR "physical activity" OR athletes |  |
|  | **N=334,272** |  |
| [3] | Sports OR sport OR performance OR "athletic performance" OR "Physical Functional Performance" OR "physical performance" OR "jump performance" OR "repeated sprint" OR sprint OR "sprint performance" OR speed OR "Muscle Strength" OR strength OR "anaerobic performance" OR "aerobic performance" OR power OR "Physical Endurance" OR endurance OR Exercise OR "high-intensity exercise" OR "repeated high-intensity exercise" |  |
|  | **N=11,411,503** |  |
| **Scopus 30/08/2022** | | |
| [1] | nap OR napping OR "daytime nap*" OR "daytime sleep*" OR siesta | **[1] AND [2] AND [3]**  **N=358** |
|  | **N=23,921** |  |
| [2] | "physically active*" OR "physical activity*" OR athletes* |  |
|  | **N=350,785** |  |
| [3] | sport* OR performance* OR "athletic performance*" OR "Physical Functional Performance*" OR "physical performance*" OR "jump performance*" OR "repeated sprint*" OR sprint* OR "sprint performance*" OR speed* OR "Muscle Strength" OR strength* OR "anaerobic performance*" OR "aerobic performance*" OR power* OR "Physical Endurance" OR endurance* OR exercise* OR "high-intensity exercise*" OR "repeated high-intensity exercise*" |  |
|  | **N=14,004,997** |  |
| **SPORTDiscus 30/08/2022** | | |
| [1] | nap OR napping OR "daytime nap" OR "daytime sleep" OR siesta | **[1] AND [2] AND [3]**  **N=111** |
|  | **N=675** |  |
| [2] | "physically active" OR "physical activity" OR athletes |  |
|  | **N=278,861** |  |
| [3] | Sports OR sport OR performance OR "athletic performance" OR "Physical Functional Performance" OR "physical performance" OR "jump performance" OR "repeated sprint" OR sprint OR "sprint performance" OR speed OR "Muscle Strength" OR strength OR "anaerobic performance" OR "aerobic performance" OR power OR "Physical Endurance" OR endurance OR Exercise OR "high-intensity exercise" OR "repeated high-intensity exercise" |  |
|  | **N=1,228,099** |  |
| **CINAHL 30/08/2022** | | |
| [1] | nap OR napping OR "daytime nap" OR "daytime sleep" OR siesta | **[1] AND [2] AND [3]**  **N=84** |
|  | **N= 1,884** |  |
| [2] | "physically active" OR "physical activity" OR athletes |  |
|  | **N=161,963** |  |
| [3] | Sports OR sport OR performance OR "athletic performance" OR "Physical Functional Performance" OR "physical performance" OR "jump performance" OR "repeated sprint" OR sprint OR "sprint performance" OR speed OR "Muscle Strength" OR strength OR "anaerobic performance" OR "aerobic performance" OR power OR "Physical Endurance" OR endurance OR Exercise OR "high-intensity exercise" OR "repeated high-intensity exercise" |  |
|  | **N=713,623** |  |
| **SCielo 30/08/2022** | | |
| [1] | nap OR napping OR "daytime nap" OR "daytime sleep" OR siesta | **[1] AND [2] AND [3]**  **N=0** |
|  | **N= 107** |  |
| [2] | "physically active" OR "physical activity" OR athletes |  |
|  | **N=8 762** |  |
| [3] | Sports OR sport OR performance OR "athletic performance" OR "Physical Functional Performance" OR "physical performance" OR "jump performance" OR "repeated sprint" OR sprint OR "sprint performance" OR speed OR "Muscle Strength" OR strength OR "anaerobic performance" OR "aerobic performance" OR power OR "Physical Endurance" OR endurance OR Exercise OR "high-intensity exercise" OR "repeated high-intensity exercise" |  |
|  | **N=95 895** |  |
| **EBSCOhost 30/08/2022** | | |
| [1] | nap OR napping OR "daytime nap" OR "daytime sleep" OR siesta | **[1] AND [2] AND [3]**  **N=189** |
|  | **N= 20,457** |  |
| [2] | "physically active" OR "physical activity" OR athletes |  |
|  | **N=650,197** |  |
| [3] | Sports OR sport OR performance OR "athletic performance" OR "Physical Functional Performance" OR "physical performance" OR "jump performance" OR "repeated sprint" OR sprint OR "sprint performance" OR speed OR "Muscle Strength" OR strength OR "anaerobic performance" OR "aerobic performance" OR power OR "Physical Endurance" OR endurance OR Exercise OR "high-intensity exercise" OR "repeated high-intensity exercise" |  |
|  | **N=12,304,206** |  |


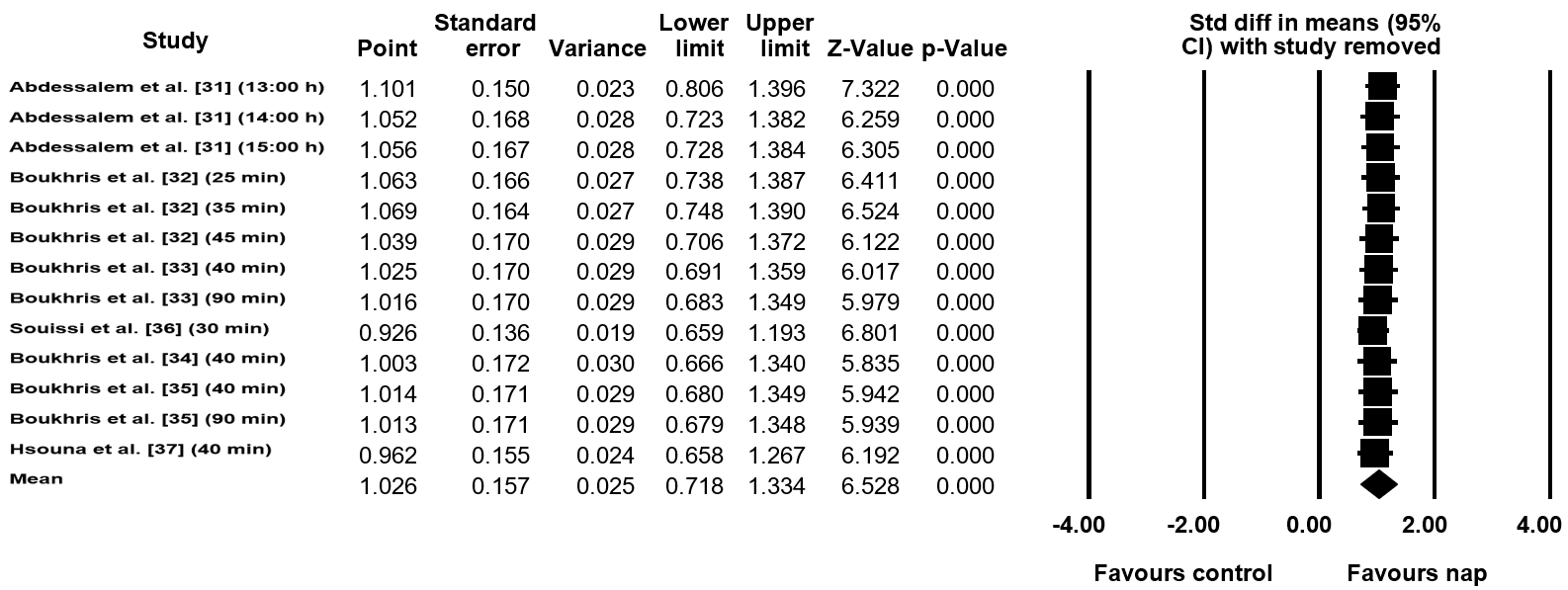


**Figure S1.** Statistic with study removed for highest distance. Std diff: standard difference; CI: confidence intervals.


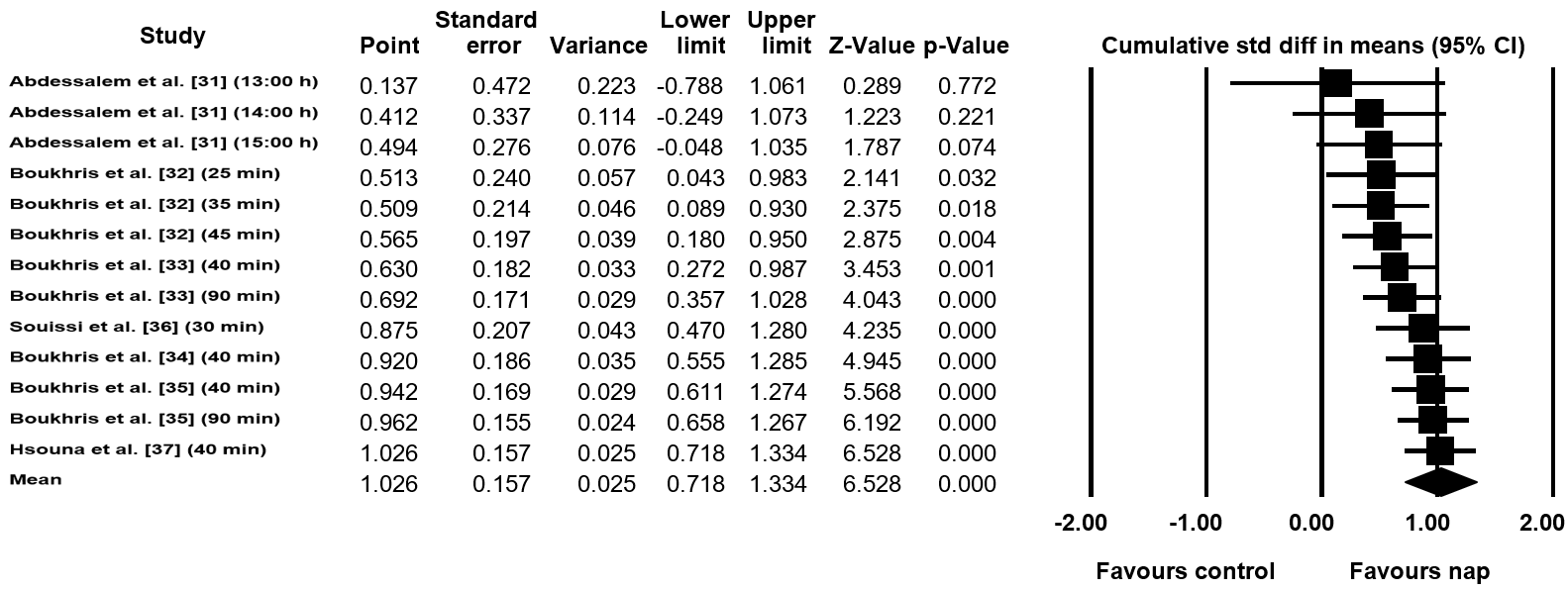


**Figure S2.** Cumulative statistics for highest distance. Std diff: standard difference; CI: confidence intervals.


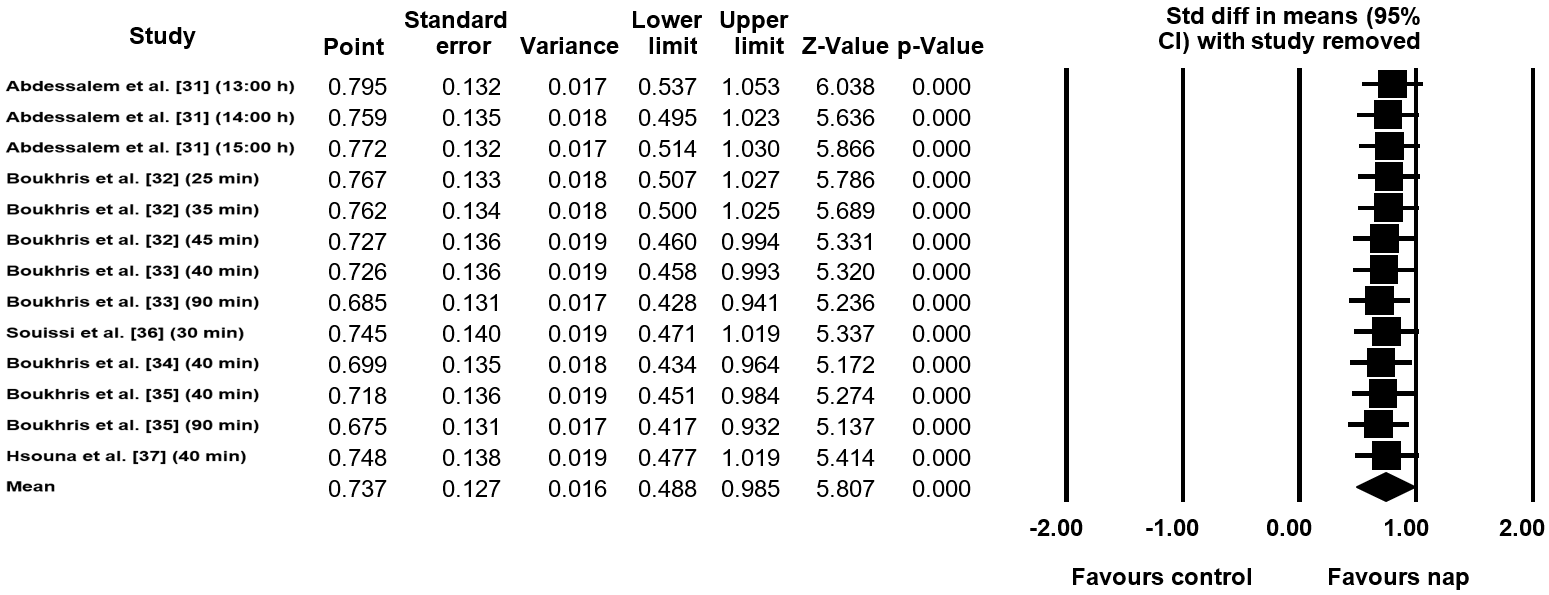


**Figure S3.** Statistic with study removed for total distance. Std diff: standard difference; CI: confidence intervals.


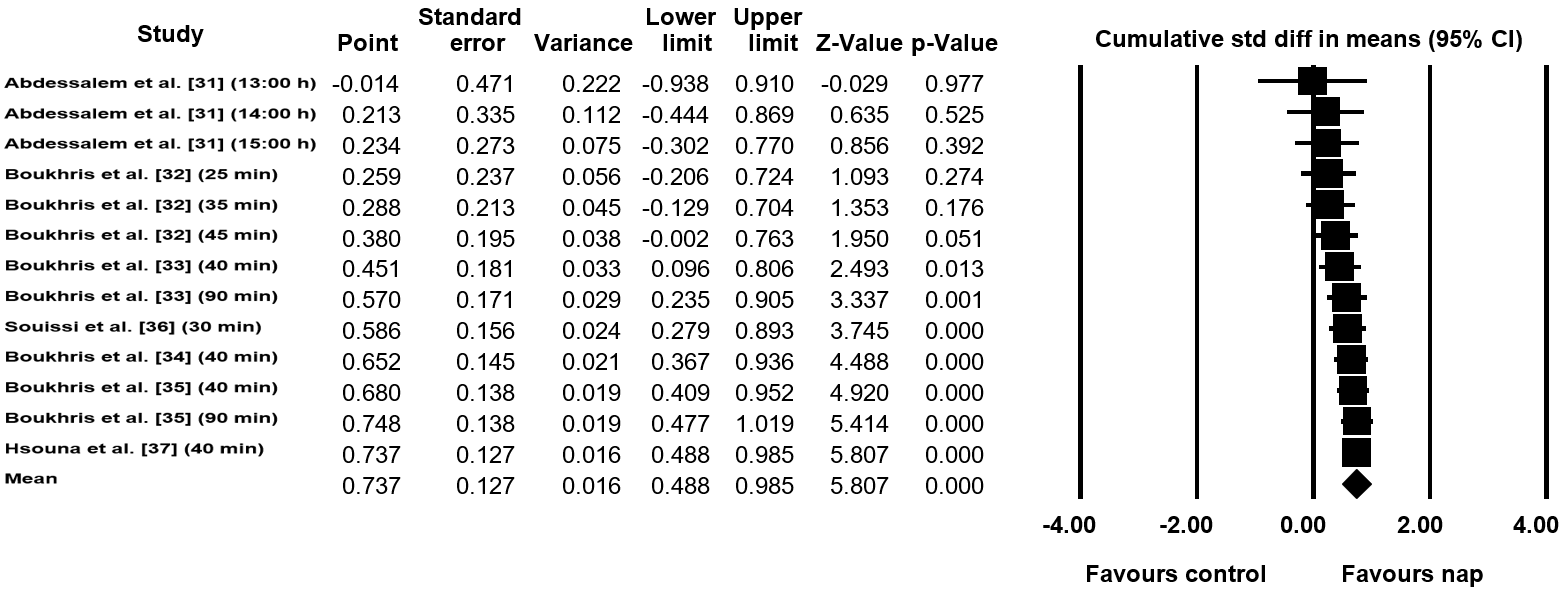


**Figure S4.** Cumulative statistics for total distance. Std diff: standard difference; CI: confidence intervals.


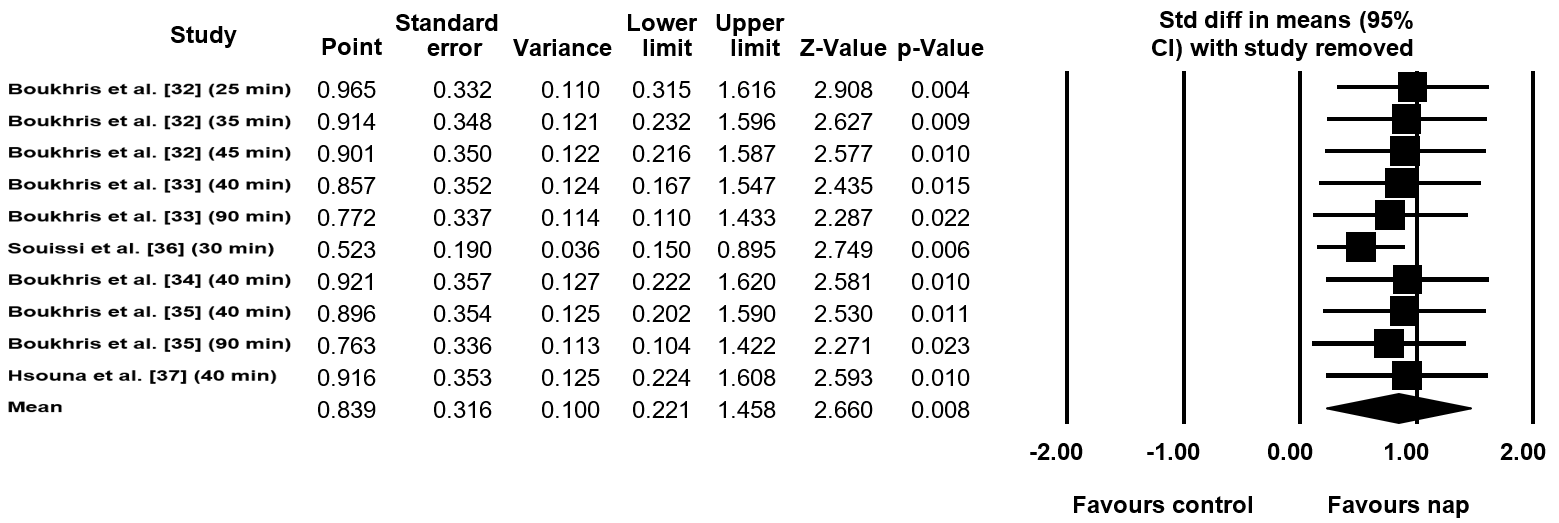


**Figure S5.** Statistic with study removed for fatigue index. Std diff: standard difference; CI: confidence intervals.


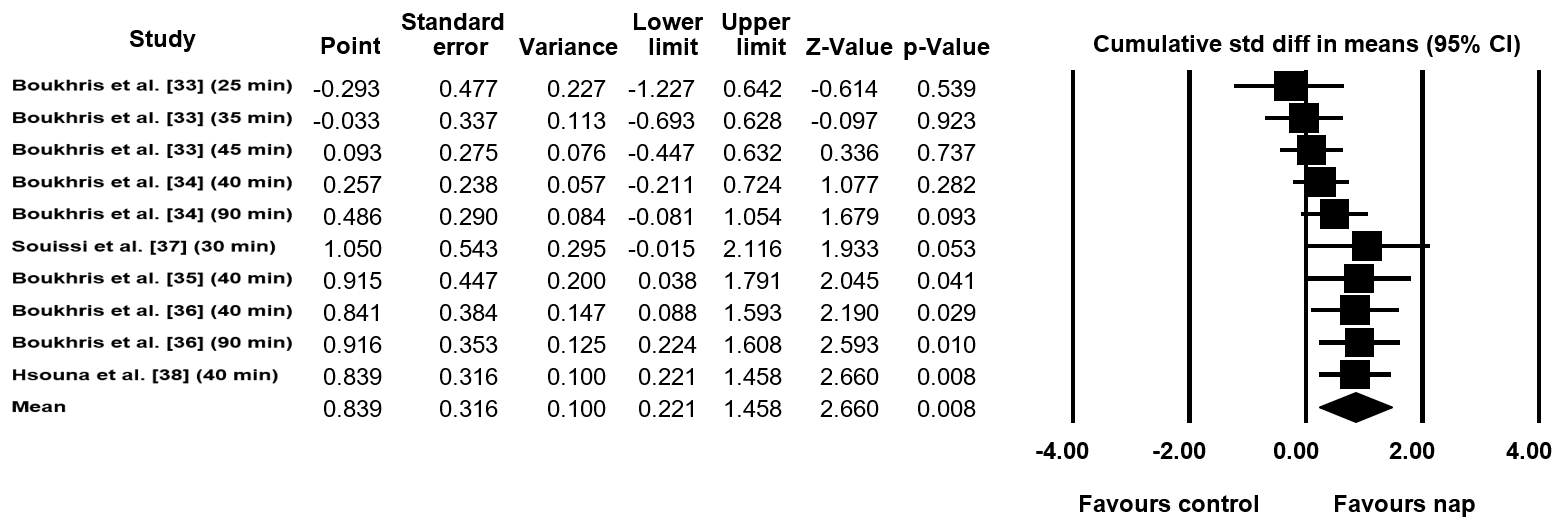


**Figure S6.** Cumulative statistics for fatigue index. Std diff: standard difference; CI: confidence intervals.


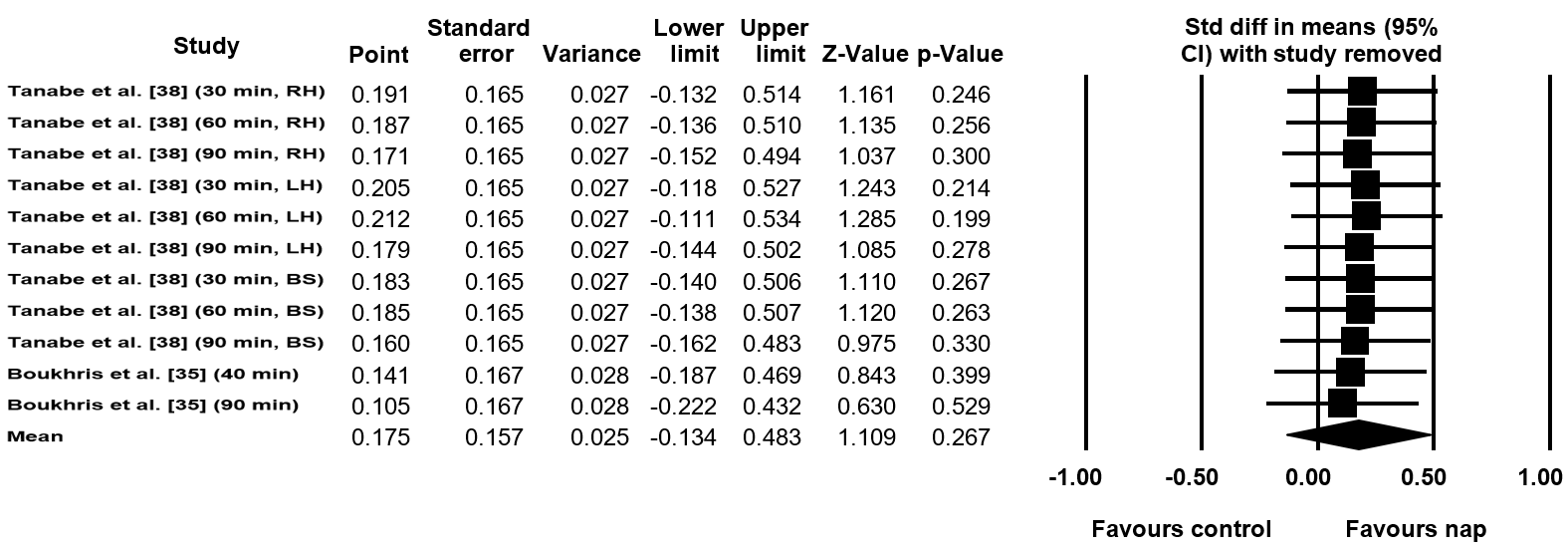


**Figure S7.** Statistic with study removed for muscle force. Std diff: standard difference; CI: confidence intervals.


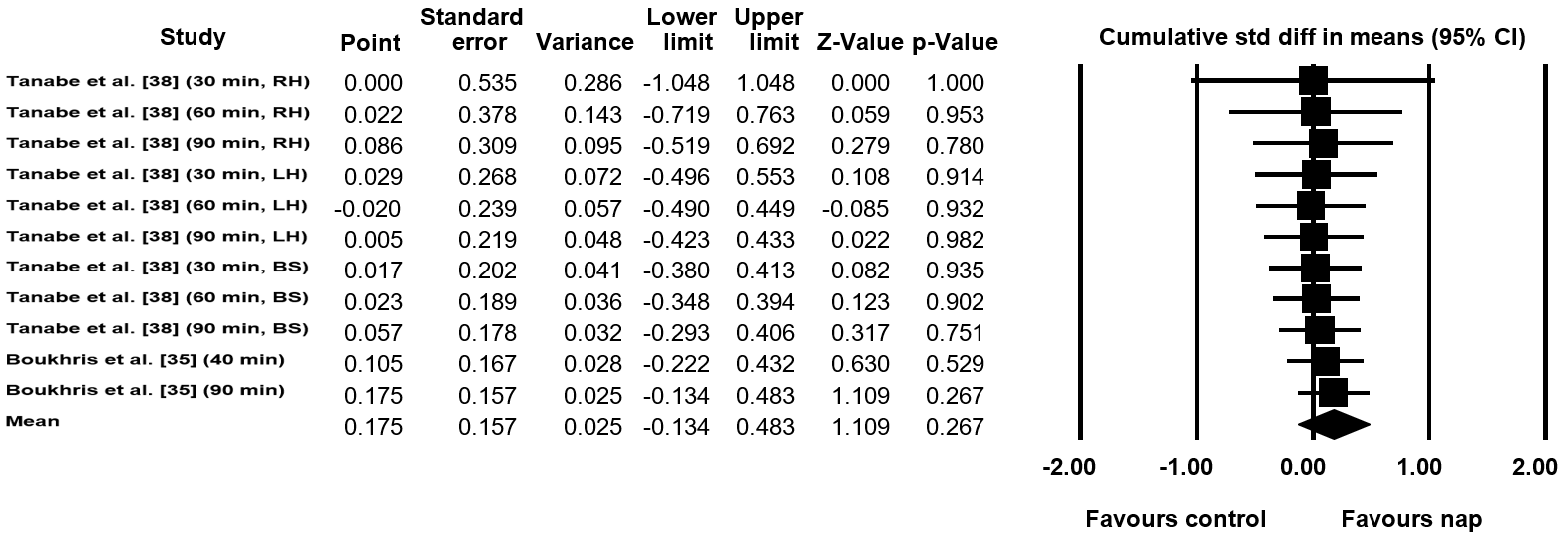


**Figure S8.** Cumulative statistics for muscle force. Std diff: standard difference; CI: confidence intervals.
